# Supplementary material for: Strengthening capacity in hospitals to reduce perinatal morbidity and mortality through a codesigned intervention package: protocol for a realist evaluation as part of a stepped-wedge trial of the Action Leveraging Evidence to Reduce perinatal morTality and morbidity (ALERT) in sub-Saharan Africa project
Source: BMJ Open. 2022 Apr 18;12(4):e057414. doi: 10.1136/bmjopen-2021-057414 (PMC9020280; doi:10.1136/bmjopen-2021-057414)
Supplement: Supplementary data [file bmjopen-2021-057414supp001.pdf]

Additional File 1

Characteristics of the study countries

	Benin	Malawi	Tanzania	Uganda
Country-level indicators				
Estimated population (in 2020, million)	12.1	20.3	62.8	47.2
Maternal mortality ratio per 100,000 live births (2015) [3]	405	634	398	343
Neonatal mortality rate per 1,000 live births (2018) [1]	32.7	22.7	21.1	20.2
Stillbirth rate per 1,000 total births (2015) [102]	30.3	21.8	22.4	21.0
% of births in health facilities (DHS Statcompiler) #	86.9%	91.4%	62.6%	73.4%
Annual growth rate in % of births in health facilities #	1.3%	3.2%	2.6%	4.4%
% of facility births in hospitals #	55.1%	42.2%	47.8%	47.8%
% of all live births by caesarean section # / Poorest v Richest wealth quintile	5.4%<br>2.7% - 11.5%	6.1%<br>3.0% - 9.1%	5.9%<br>2.4% - 15.8%	6.2%<br>2.7% - 14.2%
Among live births in health facilities				
% of women checked by a health professional before discharge from facility following childbirth #	68%	57%	51%	47%
% of all live births by caesarean section #	6.3%	6.3%	9.5%	8.3%
Among live births in hospitals				
Neonatal mortality per 1,000 live births #	20.6	35.8	31.8	27.1
% of newborns breastfed within 1 hour of birth #	67.8%	73.1%	54.9%	65.3%
Health system indicators				
Doctors /10,000 people population ^	1.6 (2016)	1.2 (2016)	0.4 (2014)	0.9 (2015)
Nursing cadres /10,000 people population ^	6.2 (2016)	2.5 (2016)	4.1 (2014)	6.3 (2015)
Predominant midwifery provider [103]	Midwife	Nurse-midwife	Nurse-midwife	Midwife
Hospital beds / 10,000 people population ^	5 (2010)	13 (2011)	7 (2010)	5 (2010)
Current health expenditure per capita (USD PPP, 2015) ^	85	108	97	139
Out-of-pocket expenditure as % of current health expenditure (2015?)^	41	11	26	41
User fees for childbirth (vaginal/caesarean) ^	Official fees	No official	No official	No official

additional analysis of Demographic and Health Survey data, Benin; 2011-12; Malawi: 2015-16; Tanzania: 2015-16; Uganda: 2016
^WHO observer http://apps.who.int/gho/data/node.main.HWFGRP_0020?lang=en
