# Supplementary material for: Strengthening capacity in hospitals to reduce perinatal morbidity and mortality through a codesigned intervention package: protocol for a realist evaluation as part of a stepped-wedge trial of the Action Leveraging Evidence to Reduce perinatal morTality and morbidity (ALERT) in sub-Saharan Africa project
Source: BMJ Open. 2022 Apr 18;12(4):e057414. doi: 10.1136/bmjopen-2021-057414 (PMC9020280; doi:10.1136/bmjopen-2021-057414)
Supplement: Author's manuscript [file bmjopen-2021-057414.draft_revisions.pdf]

BMJ Open is committed to open peer review. As part of this commitment we make the peer review history of every article we publish publicly available.

When an article is published we post the peer reviewers' comments and the authors' responses online. We also post the versions of the paper that were used during peer review. These are the versions that the peer review comments apply to.

The versions of the paper that follow are the versions that were submitted during the peer review process. They are not the versions of record or the final published versions. They should not be cited or distributed as the published version of this manuscript.

BMJ Open is an open access journal and the full, final, typeset and author-corrected version of record of the manuscript is available on our site with no access controls, subscription charges or pay-per-view fees (http://bmjopen.bmj.com).

If you have any questions on BMJ Open's open peer review process please email info.bmjopen@bmj.com

BMJ Open

Action Leveraging Evidence to Reduce perinatal morTality and morbidity in sub-Saharan Africa (ALERT): the protocol of a realist evaluation

Journal:	*BMJ Open*
Manuscript ID	bmjopen-2021-057414
Article Type:	Protocol
Date Submitted by the Author:	16-Sep-2021
Complete List of Authors:	Abejirinde, Ibukun-Oluwa; Hospital for Sick Children, International Program Evaluation Unit, Centre for Global Child Health ; University of Toronto Dalla Lana School of Public Health, Division of Social & Behavioural Health Sciences<br>Castellano Pleguezuelo, Virginia; Institute of Tropical Medicine<br>Benova, Lenka; Institute of Tropical Medicine, Department of Public Health<br>Dossou, Jean-Paul; Centre de Recherche en Reproduction Humaine et en Démographie<br>Hanson, Claudia; Karolinska Institute, Dept Public Global Health<br>Metogni, Christelle Boyi; Centre de Recherche en Reproduction Humaine et en Démographie<br>Meja, Samuel; University of Malawi College of Medicine<br>Mkoka, D; Muhimbili University of Health and Allied Sciences<br>Namazzi, Gertrude; Makerere University, Department of Health Policy Planning and Management<br>Sidney, Kristi; Karolinska Institute, Global Public Health<br>Marchal, Bruno; Institute of Tropical Medicine, Department of Public Health
Keywords:	EDUCATION & TRAINING (see Medical Education & Training), Clinical governance < HEALTH SERVICES ADMINISTRATION & MANAGEMENT, Quality in health care < HEALTH SERVICES ADMINISTRATION & MANAGEMENT, OBSTETRICS, PERINATOLOGY

SCHOLARONE™
Manuscripts

Action Leveraging Evidence to Reduce perinatal morTality and morbidity in sub-Saharan Africa (ALERT): the protocol of a realist evaluation

Ibukun-Oluwa Omolade Abejirinde[1], Virginia Castellano Pleguezuelo[2], Lenka Benova[2], Jean-Paul Dossou[3], Claudia Hanson[4,5], Christelle Boyi Metogni[3], Samuel Meja[6], Dickson Mkoka[7], Gertrude Namazzi[8], Kristi Sidney Annerstedt[4] and Bruno Marchal[2]

Affiliations

[1] Social and Behavioural Health Sciences Division, Dalla Lana School of Public Health, University of Toronto, Toronto (Canada)

[2] Department of Public Health, Institute of Tropical Medicine, Antwerp (Belgium)

[3] Centre de Recherche en Reproduction Humaine et en Démographie (CERRHUD), Cotonou, Benin

[4] Department of Global Public Health, Karolinska Institutet, Sweden

[5] Department of Disease Control, London School of Hygiene and Tropical Medicine, London, United Kingdom

[6] College of Medicine, The Centre for Reproductive Health, University of Malawi, Blantyre, Malawi

[7] Department of Obstetrics and Gynaecology, Muhimbili University of Health and Allied Sciences, Dar Es Salaam, Tanzania

[8] Centre of Excellence for Maternal Newborn and Child Health, Department of Health Policy Planning and Management, School of Public Health, Makerere University, Uganda

For peer review only

Corresponding author

Bruno Marchal, Department of Public Health, Institute of Tropical Medicine, Antwerp

Nationalestraat 155, 2000 Antwerp, Belgium

bmarchal@itg.be

+32.2476384

For peer review only

Abstract

Introduction

Despite a strong evidence base for developing interventions to reduce child mortality and morbidity related to pregnancy and delivery, major knowledge-implementation gaps remain. The Action Leveraging Evidence to Reduce perinatal morTality and morbidity in sub-Saharan Africa (ALERT) project aims to overcome these gaps through multidisciplinary teams that provide maternity care. This paper presents the research protocol of the realist process evaluation of the ALERT intervention.

Methods and analysis

Following the realist evaluation cycle, we will first elicit the initial programme theory on the basis of the ALERT theory of change, a review of the evidence on adoption and diffusion of innovations, and the perspectives of the stakeholders. Second, we will use a multiple embedded case study design to empirically test the initial programme theory in two hospitals in each of the four intervention countries. Qualitative and quantitative data will be collected. Analysis will be guided by the Intervention-Actors-Context-Mechanism-Outcome configuration heuristic. We will use thematic coding to analyse the qualitative data. The quantitative data will be analysed descriptively and integrated in the analysis using a retroductive approach. Each case study will end with a refined programme theory (in-case analysis). Third, we will carry out a cross-case comparison within and between the four countries. Comparison between study countries should enable identifying relevant context factors that influence effectiveness and implementation, leading to a mid-range theory that may inform the scaling up the intervention.

Ethics and dissemination

In developing this protocol, we paid specific attention to cultural sensitivity, the do no harm principle, confidentiality and non-attribution. We received ethical approval from the local and national institutional review boards in Tanzania, Uganda, Malawai, Benin, Sweden and Belgium.

For peer review only

Strengths and limitations

- In quality improvement interventions, variation in results may be explained by the interplay between implementation process, context and agency of the actors.
- The MRC guidance on process evaluation calls for focusing evaluations on the intervention theory, but studies doing so remain rare.
- Realist evaluation enhances the explanatory power of process evaluations by (1) providing an analytical approach that starts from an intervention theory and explains the outcome in terms of a configuration of intervention, context, actors and mechanisms of change, and (2) by testing and developing the theoretical basis of implementation and scaling up of effective interventions.
- Study limitations include potential risks presented by cross-cultural differences, power imbalances and western-dominated theories in realist research

Key words

Process evaluation, realist evaluation, programme theory, theory-testing, quality improvement

For peer review only

Introduction

Child mortality and mortality related to pregnancy and childbirth remain major public health priorities. Globally, five million children die before their second birthday[1] and nearly 7.5 million children die within the first 1000 days of life. Over two million babies are stillborn each year[2] and for almost 300,000 women annually, pregnancy or childbirth are fatal.[3]

While there is strong evidence available related to what ought to be made available to women and their new-borns during childbirth[4], there is a lack of evidence on how best to support health providers to implement the evidence-based practices which can save lives and prevent suffering. The Action Leveraging Evidence to Reduce perinatal morTality and morbidity in sub-Saharan Africa (ALERT) project was developed to close the gap between evidence and practice. ALERT is a 5-year multifaceted health system intervention targeting the intrapartum period. It is funded by the European Commission under the Horizon 2020 framework and led by Karolinska Institute in collaboration with seven other institutions. National universities and health care professional organisations in cooperation with local district health management teams and local training institutions will deliver the ALERT intervention.

The project aims to (1) overcome the knowledge-implementation gap at the level of multidisciplinary teams providing maternity care and (2) promote implementation of evidence-based interventions in hospitals with a target primary outcome of reduced in-facility perinatal (stillbirths and early neonatal) mortality. The ALERT project will develop an intervention that includes four main components (Figure 1):

1. Co-design of the intervention via end-user participation, involving narratives of women, families and midwifery providers
2. Competency-based training for midwives
3. Quality improvement cycles to help providers identify and overcome problems, supported by data from a clinical perinatal e-registry

For peer review only

4. Mentoring maternity unit leaders to foster empowerment and leadership, complemented by bi-annual coordination and accountability meetings at district level.

The intervention is based on a framework that builds on many years of experience working in the respective settings and the literature on quality improvement and learning (including, for instance, the work of Rowe *et al.*[5] and Walker *et al.*[6] It was deliberately designed to integrate four different improvement strategies to support health care workers in providing safe and respectful intrapartum care. It integrates the perspectives of midwifery providers as well as women and their families. The latter will be involved in co-designing the training and QI intervention. The intervention was field tested during a visit to a regional hospital in Dar-es-Salaam, Tanzania in February 2019. The intervention will integrate QI and in-service training explicitly as one approach and thus leverage previous experience suggesting that in the ALERT settings knowledge deficits remain an important bottleneck and may create better engagement into QI. [6 7] Mentoring by valued mentors, an essential aspect of QI [8], will be carefully designed and include a cascade approach to meet the needs of the health care workers.

Figure 1 - The ALERT intervention

The implementation started in Benin, Malawi, Tanzania and Uganda in 2020 and will run until 2024, steered by seven scientific work packages (WPs). Four district-level hospitals were selected in each country, representing a mix of public and private not-for-profit facilities. The intervention will be rolled out as indicated in Figure 2. The hospitals were randomly selected for inclusion (Figure 2). More background information for each country is provided in Additional file 1.

Figure 2 - Sequential integration of hospitals into the trial

An implementation science approach is at the heart of this study. Since the implementation of evidence-based guidelines (and arguably any quality improvement strategy) is not only determined by providers, funding and health care system capacity [9] but also by its adoption and implementation by individuals and teams, it is critical to understand what works and why.[10 11] Three approaches will be employed to evaluate the ALERT intervention:

i. A stepped-wedge design to evaluate the effect of the intervention on in-facility perinatal mortality with a primary outcome of reduced in-facility perinatal (stillbirths and early neonatal) mortality

ii. A realist process evaluation to understand what works, for whom, and under which conditions, complemented by an economic evaluation

iii. Economic evaluation focused on scalability including costing studies in the countries.

In this paper, we present the research protocol of the realist process evaluation. Process evaluations (PE) are often carried out in implementation research, not only to address the issue of the black box between an intervention and its results, but also because they are considered as useful in the design and testing of complex interventions.[12] A process evaluation considers the actual implementation of the programme, the underlying mechanisms of change and the effect of context on outcomes.[13] To this end, process evaluations often incorporate an assessment of implementation (fidelity), a stakeholder analysis, and a context mapping. We developed the process evaluation on the basis of realist evaluation because of its explanatory potential: realists assume that interventions lead to effects in specific contexts, by triggering mechanisms of change for specific groups of actors. This configurational approach to causation allows for greater in-depth understanding of why and for whom intervention work (or not).[14] Over the years, RE has become recognized in health policy and systems research as a useful methodology for evaluating complex interventions.[15 16 17] This study contributes to filling a gap, as few studies in the field of implementation science have adopted the realist evaluation approach. We

For peer review only

followed the RAMESES II guidance for reporting on realist evaluations[18], which we have adapted to fit the format for a protocol paper (see Additional file 2).

Methods and analysis

The overall methodological approach

In the quest to open the 'black-box' of complex programmes, Pawson and Tilley developed realist evaluation (RE), a theory-driven research approach that seeks to understand the links between context, outcomes and mechanisms.[19] While traditional evaluation questions often focus on effectiveness or on process, RE seeks to answer a string of questions: *what works, for whom, in what respect, to what extent, in what context and how?*

From the perspective of RE, programmes work (or not) because actors make decisions in response to the opportunities or resources that the programme provides. According to Pawson and Tilley, programmes or interventions can be viewed as theories or assumptions held by people (including designers, implementers, beneficiaries, and policymakers) who are embedded in the open social systems in which they operate. Therefore, it is not the programme, but the actors who are engaged in it who bring about the results. To understand how that happens, RE explores the context in which the programme operates and the mechanisms that are underlying the observed change (or the lack thereof). Mechanisms refer to psychological, social, cultural or organisational factors that explain why actors respond to a programme the way they do. This response, however, does not happen in isolation; the context in which the programme takes place influences the way actors respond to it: mechanisms (and thus the programmes that trigger them) only work if the context conditions are right. Summarily, not every programme works identically in different settings.

Realist evaluators use the term *programme theory (PT)* to describe the explanation of how a programme has contributed to observed results. The programme theory is not only the end product of a realist

For peer review only - http://bmjopen.bmj.com/site/about/guidelines.xhtml

evaluation, but it is also a hypothesis that serves as the starting point of any realist evaluation. The initial PT shapes the study design, data collection, analysis and synthesis.

We structured the process evaluation of ALERT according to the steps of the realist research cycle[20] (figure 3).

- In consideration of the topic being researched, realists build the **initial PT** upon existing evidence (e.g. literature reviews, document reviews), interviews with key actors and/or exploratory research.
- The **study design** is chosen as a function of the programme theory that needs to be assessed.
- **Data collection** tools and methods are selected based on the components of the initial programme theory to be tested: all data required to test the initial PT should be collected. Often mixed methods (qualitative and quantitative) of data collection are used.
- **Data analysis**: the Intervention-Context-Actor-Mechanism-Outcome (ICAMO) configuration heuristic is often used in the analysis of data.[14] Qualitative data analysis is usually based on a thematic coding approach using the core elements of the initial PT. The quantitative data are often analysed with descriptive statistics and integrated in the analysis using a retroductive approach.
- Each study ends with a **synthesis** based on a comparison of the findings of the study with the initial PT, which is adapted as needed, leading to a refined PT.

Figure 3 – The realist research cycle (source: [20])

For peer review only

Objectives

The main objective of the ALERT process evaluation is to identify and test the causal pathway through which the intervention achieves its impact. Summarily, it aims to answer the question *"Which components of the ALERT intervention work, how, for whom and in which contexts?"*

There are four specific objectives:

i. To collaboratively elicit the initial programme theory of the ALERT intervention

ii. To test the initial programme theory through a multiple embedded case study

iii. To refine the programme theory of the ALERT intervention using cross-case analysis

iv. To summarise and disseminate findings in the form of recommendations to inform implementation and practice (of quality intrapartum care) at hospital and country levels.

In the following sections, each of the four core objectives and the corresponding data collection and analysis methods are presented.

Objective 1: Defining the Initial Programme Theory

We will formulate the initial ALERT PT based on its Theory of Change (TOC) (see Figure 4), a review of the existing evidence on adoption and diffusion of innovations, and the perspectives of stakeholders involved in the intervention.

ALERT's theory of change is informed by the integrated Promoting Action on Research Implementation in Health Services (i-PARIHS) framework.[21] It identifies the *what* (i.e. an intervention that fits with existing practice, is user-friendly, has a comparative advantage and can be tested); the *who* or *recipients* (i.e. end users: midwifery providers, mothers and their families, who are motivated and have sufficient skills and resource support by collaboration and teamwork); the *where* or *context* (sub-Saharan African (SSA) hospitals with formal and informal leadership support, effective evaluation and feedback

For peer review only - http://bmjopen.bmj.com/site/about/guidelines.xhtml

processes and a conducive learning environment) and the *how* (i.e. a problem-focused multi-faceted approach to ensure participation and involvement, ownership and control, and empowerment within and between groups).

The TOC implies that end-user participation ensures that the needs of women, families and midwifery providers (i.e. the end users) are identified and addressed in the form of training and quality improvement (QI). Also, better trained and supported midwifery providers will provide better care, including improved foetal monitoring, improved emergency care, and client-centred support during labour. Furthermore, better skilled and empowered midwifery providers will also be better able to quickly make decisions. Finally, improved hospital processes leading to timely access to caesarean section or operative vaginal delivery will reduce hypoxic-ischaemic events and thus reduce stillbirths and neonatal deaths.

Figure 4 – The ALERT theory of change

In order to move the TOC to the level of an initial programme theory, we would need to identify the context elements and mechanisms that would explain how and why the ALERT intervention would achieve the expected results. We plan to carry out the following steps:

- We will summarise existing evidence to identify any theories that may explain how and in which conditions the ALERT intervention will be effective. More concretely, we will focus on the adoption, diffusion, and maintenance of the intervention (components) by health care providers and hospital managers, and the role of multi-stakeholder co-production in these processes.

For peer review only

- We will draw upon health facility assessment data, stakeholder mapping data and the national context assessment data collected by other work packages in the intervention, to describe the national and facility context, and the views and perspectives of national stakeholders.
- We will organise participatory stakeholder workshops to elicit the assumptions of the ALERT consortium members.
- We will use the opportunity of online and face-to-face monthly coordination meetings, consortia meetings, discussions and feedback sessions to present and discuss the evolving initial programme theory to our colleague-researchers and stakeholders from each country.

The end product of these is the initial programme theory that will guide the further steps of the process evaluation.

Objective 2: A multiple embedded case study to test the initial PT

To test the initial PT, we will use a case study design. Investigating the intervention's implementation through case studies in hospitals in four countries will allow us to monitor the actual implementation of the intervention and how it progressively evolves (in terms of actual implementation, context conditions, response of actors and potential mechanisms) across the study sites.

The case study design

We will carry out a multiple embedded case study.[22] We define 'the case' as the implementation of the ALERT intervention. We define 'study site' as a hospital implementing the ALERT intervention. In realist evaluation, study sites are chosen purposively in order to allow testing the initial PT (i.e. theoretical sampling). In practice, in each country, we will select two of the four study sites, whereby we aim to choose contrasting sites that are likely to 'manifest' the IPT differently due to contextual variation.

In each country, the first site selected for the realist process evaluation is the hospital where the ALERT intervention will start at step 1 (Month19 - M24) of the stepped wedge intervention design (Figure 2).

For peer review only

In these four hospitals, we will conduct a detailed “thick” evaluation (see details on data collection below). The second hospitals will be chosen among the stage 3 hospitals (M31 - M36 of implementation), and a “light” evaluation will be conducted. The phased recruitment of hospitals into the realist process evaluation will enable the assessment of how length of exposure to the intervention and changes over time influence outcomes. Selecting two hospitals per country will allows us to conduct in-country cross-case comparisons and to identify how mechanisms play out differently at country and facility levels.

Data collection

We will use the following data collection methods and tools:

- In each site, we will carry out in-depth interviews with health stakeholders (including midwives, maternity unit managers, hospital managers, the district director of health), focusing on their view on the reasons the ALERT intervention works (or not).
- This will be complemented by opportunistic observations and review of relevant hospital documents.
- We will draw upon data collected by other work packages to describe the actual implementation of the intervention, as well as the context, the actors and the underlying processes.
- We will carry out in-depth interviews with mothers and their families to document their views on the design and implementation of the ALERT intervention.
- We will draw upon the data from patient exit interviews carried out by another ALERT work package to obtain additional information on the views of patients on the care they received and the (potential) influence of the ALERT intervention.

Intervals between data collection offer time to analyse data and refine the IPT based on emerging patterns which will be presented at the subsequent consortium meetings. Table 1 summarises the data collection plan.

For peer review only

Table 1. Summary of ALERT Realist process evaluation data collection

**Specific objective**	**Data collection methods**	**Product/finality**
Defining the Initial Programme Theory	i. Scoping review<br>ii. ALERT Consortium stakeholder workshop<br>Iii. Country level stakeholder workshops (x4)<br><br>Data used from other work packages<br>i. Health facility assessments and Bottleneck analysis<br>ii. Implementation process description	The initial programme theory of the ALERT intervention
Multiple embedded case study to test the IPT	i. Context mapping (hospital)<br>ii. Context mapping (national/health system)<br>iii. In-depth interviews with women and their families<br>iv. In-depth interviews with midwives, maternity unit managers, hospital managers, district directors of health<br><br>Data used from other work packages<br>i. Review data collection by WP 2 and 3 from narratives and ethnographic observations of women and health providers	Site-specific ICAMO configurations and potentially programme theories
Cross-case analysis to refine the programme theory of ALERT	This step involves first the comparison of the country-specific programme theories in order to identify patterns of similarities and differences.<br>In a second step, the findings are brought to a next level of abstraction, aimed at capturing the essential elements that explain the results of each site in a comprehensive manner	i. Country-specific programme theories<br>ii. Overarching programme theory

Data collection tools

For each data collection method described above, specific tools will be used: a facilitator guide for the participatory stakeholder workshops for ALERT consortium members; in-depth interview guides for hospital managers/in-charges, midwives and district directors of health; and in-depth interview guides for mothers and families (the latter interviews will be carried out within another work package). Local research assistants will collect data in each country. To bridge gaps in technical capacity, especially with

For peer review only

respect to realist interviewing techniques [23], we will conduct face-to-face training, complemented by remote training and support.

Data management

Audio files (and handwritten notes) will be translated, transcribed verbatim, and managed for analysis using NVIVO© qualitative data analysis software. Translation will aim to retain technical and cultural meaning in order to minimise interpretation gaps resulting from cross-cultural differences.[24] A random selection of audio files and their corresponding transcripts will be reviewed and checked for quality and accuracy by a member of the realist process evaluation team. Audio files and interview transcripts will be tagged alpha-numerically based on date, district identifier and category of stakeholder. Individuals will not be identified by their real names in field notes. After each data collection session, all information (raw and transcribed data) will be stored on password-protected devices (external drives, computers) and will only be accessed by evaluation team members.

Data analysis

As mentioned above, RE is method-neutral: discipline-specific and appropriate techniques are adopted during data analysis. In general, the analysis will be guided by the ICAMO configuration. Analysis of the qualitative data will be guided by the primary elements and themes identified in the initial ALERT programme theory. Analysis of transcripts will begin immediately following transcription, allowing for early identification of ICAMO configurations and patterns. In line with realist principles, a thematic coding approach will be used that is based on the core elements of the initial PT. The framework analysis method [25] suits the realist approach as it allows for the inclusion of both a priori and emergent concepts.

- The data will be categorised in a first round of analysis using the ICAMO configuration.
- New interpretations will emerge in subsequent rounds of coding, leading to a refined analysis.
- Next, a retroduction approach will be adopted, whereby the observed outcomes are explained by looking into the mechanisms, actual intervention modalities, actors and context elements. This results in descriptions of the actual intervention, its effects (both intended and unintended,

For peer review only

positive and negative), the context elements and the underlying mechanisms (ICAMO configurations), which offer the most plausible explanations of the observed outcomes.

During this analytical process, we will also draw upon data and findings from other work packages and integrate participatory reflection and feedback from the ALERT consortium annual meetings. Data triangulation and verification will be ensured by comparing responses from different respondents and from other data sources in order to identify similar and divergent views. This will contribute to the internal validity of the analysis.

Synthesis

The site-specific ICAMO configuration(s) will be compared with the initial PT, and in a process of abstraction, the initial PT will be modified when and where needed.

Objective 3: Cross-case analysis to refine the programme theory of ALERT

The above described "in-case" analysis will be followed by cross-case analysis within and between the countries. Comparing between the study countries should enable identifying relevant context factors at country level and assessing their influence on intervention implementation and scale up.

In practice, we will develop tables presenting the different ICAMO configurations by country. We will look for patterns of similarity and difference and see how these challenge, refute or confirm the initial programme theory. Prior to the finalisation of the refined programme theory, the results of the process evaluation will be discussed in a final round of participatory workshops with ALERT partners and in-country stakeholders directly involved in the project. This will provide an opportunity to contextualise, reflect on and improve the programme theory.

In summary, the final round of PT development will occur iteratively during the cross-case analysis, leading to a final programme theory that may reach the level of a mid-range theory, which indicates what it is about the ALERT intervention that works for whom and in which circumstances. The results

For peer review only

may not only inform the formulation of recommendations for scaling up the intervention in the study countries, but may also allow tailoring of similar interventions to different contexts.

Objective 4: Dissemination

To summarise and disseminate findings in the form of recommendations at study site and country level, we will leverage the annual consortium project meetings. This also serves as an opportunity for cross-pollination of experiences and learning between intervention countries, and for presenting and discussing the research findings within the broader findings of the project. Feedback meetings will adopt a participatory open discussion approach with the aim to discuss potential and critical action points for improving implementation and scaling up to other locations, as well as the possible implications (positive and negative) of such efforts.

In addition to the final country reports on the RE from each country, findings will be further disseminated through at least two open-access publications and two international conferences. We expect that the doctoral student working on this process evaluation will provide additional avenues for dissemination at country and international level (conferences and peer-reviewed publications).

Patient and Public Involvement

In this component of the ALERT project, which in essence is an evaluation of a quality improvement intervention, patients nor members of the public were involved in the design and they will not be involved in the actual evaluation and analysis. However, during the formative phase of the project, the ALERT intervention will be co-designed with women and families living in the study sites. Their narratives regarding pregnancy, birth and their health seeking behaviour in general will be documented and analysed in order to inform the intervention design. They will be included in feedback meetings at the end of the project.

Ethics and dissemination

For peer review only

The research team recognizes the ethical implications of any form of research. The realist process evaluation will be carried out in line with the principles of the Declaration of Helsinki as amended in 2013, and any further versions.[26]

General principles

The study team will place particular emphasis on:

- *Cultural sensitivity:* Data collection modalities will be aligned to the socio-cultural norms and preferences of people in their daily life and within organizational settings (for example, ensuring interviews with health workers does not disrupt health service provision and other duties). All personnel on the study team will be bound to the code of conduct and ethical restrictions of the research.
- *Do no harm:* Respondents will be well informed of their rights to withdraw from the study at any point in time and all efforts will be made to ensure that no discomfort or stress is experienced by respondents as a result of the research. This includes allowing respondents to choose their preferred location and time for interviews with care taken to ensure privacy to the greatest degree possible. Informed consent, privacy and confidentiality, as well as respect for participants' time will be prioritized.
- *Confidentiality and non-attribution:* Only ALERT team members will have access to raw data. All documents, completed questionnaires and audio recordings will be treated with the strictest care for confidentiality. Reports will be compiled with the intention to protect the identity of respondents while representing their views and opinions as accurately and fairly as possible.

Vulnerability of the target populations

The views and experiences of respondents for the realist process evaluation are primarily collected through participatory sessions and in-depth interviews (i.e. qualitative methods). We consider that the issues to be discussed in interviews are of a non-sensitive nature and we do not foresee any harmful risks or adverse outcomes (principle of non-maleficence).

For peer review only

Due to the nature of health workers' jobs, interviews will be conducted at a place and time of their convenience without hindering their service delivery tasks. Respondents will, however, be making a sacrifice of their time in order to participate in the interviews. They will be compensated in line with the country standards for transportation re-imbursement and provision of refreshments (water, non-alcoholic beverages, and snacks).

Informed Consent

Written or verbal consent of respondents will be secured after explaining the purpose, potential benefits and potential harms of the study using an information sheet. Copies of informed consent will be available in the local language of the participants (French, Kiswahili or Chichewa) and where applicable, English. Data collectors will explain the informed consent form and respondents will be encouraged to ask for clarification or ask any questions. Respondents able to read and write will be required to sign the consent form themselves. In case of illiterate study participants, the data collectors will read the form carefully to them and they will countersign the form using their thumb impression. Where participants prefer to provide verbal consent in place of written consent, the consent will be audio-recorded. In this case, the participant or the researcher will read the paragraph granting consent aloud while being audio-recorded. Interviews conducted under verbal consent can only proceed if there is at least an audio recording of the consent. The respondent can still decline audio recording for the full interview. Where the respondent does not give consent for audio recording, only handwritten notes will be taken during the interview.

Process of withdrawal from the study

The consent procedures make it clear to research participants that they have the right to withdraw from the study at any point, and to refuse to answer questions without any negative consequences.

Possibilities for study withdrawal would include

- Where respondents have identified that they would be at risk from participating in the study

For peer review only

- Where a respondent self-reports that there has been a threat, verbal abuse or attempts to restrict their access to care or other services due to their participation in the data collection.
- When the research team identifies that there has been an incident, where recriminations have been levelled against respondents due to their participation in the study.
- Where a potential respondent declines providing any type of documented consent (written or verbal).

We refer to the last section of the Methods and analysis chapter for a discussion on the dissemination of the results of this study.

Discussion

In implementation science, finding heterogeneity in results across different settings and types of organisations and by different actors is common. This variation may often be explained by the dynamic interplay between the intervention, its context, the implementation process and the agency of involved actors. When these interactions are not accounted for in an evaluation, the result is a 'black box' of knowledge that provides little or no explanatory insight on causal factors that explain how interventions lead to outcomes. In response to the evaluation approaches dominant in the 1970s and which focused almost exclusively on effectiveness, theory-driven inquiry methods were developed.[27] Theory-based evaluation [28], theories of change [29] and realist evaluation [19] all present methods to open the black box between intervention and outcome.

The 2015 guidance on the conduct of process evaluation developed by the UK Medical Research Council (MRC) calls for paying more attention to the intervention theory.[30] This resonates with a similar shift in the field of implementation research, which is increasingly using conceptual models, frameworks and theories to design interventions but also to guide the design of evaluations, interpret findings and enhance translation of research into policy and practice. [31] Kislov *et al.* call for using mid-range theories

For peer review only

of implementation, referring to Merton's definition of middle range theories as theories that are situated between minor working hypotheses (nowadays often referred to as programme theories) and the all-inclusive unified theory (or grand theories).[32] Yet, the use of theory in process evaluations remains limited. In their recent systematic review of process evaluations embedded in trials, McIntyre and colleagues found that in about 66% of the reviewed papers, the authors stated to use theory. Yet only in 26% of the reviewed studies, theoretical propositions are actually applied, and in only 7% of the studies did the authors test theory.[33]

Previous studies have shown that realist principles can be adapted and applied to all phases of the PE cycle.[34] A realist process evaluation may offer two major advantages. First, it offers the possibility to systematically test the intervention theory by grounding it in the existing body of knowledge and testing it empirically, augmenting its explanatory power in the process.[35] Second, it allows to build up theories by accumulating empirical insights that push the programme theory to the level of a mid-range theory. Such mid-range theory has explanatory power extended to other settings, thereby offering the possibility to inform decisions on introducing or scaling up similar interventions. [36] Within the ALERT project, carrying out a realist process evaluation across eight hospitals in four countries will allow us not only to better understand the differential effects of the ALERT intervention in different hospitals in different countries, but also to contribute to the methodological developments within the field of process evaluations.

For peer review only

References

1. UN IGME. Levels & Trends in Child Mortality. Report 2020. New York: UN Inter-agency Group for Child Mortality Estimation (UN IGME), 2018.
2. GBD Child Mortality Collaborators. Global, regional, national, and selected subnational levels of stillbirths, neonatal, infant, and under-5 mortality, 1980-2015: a systematic analysis for the Global Burden of Disease Study 2015. *Lancet* 2016;388(10053):1725-74. doi: 10.1016/S0140-6736(16)31575-6 [published Online First: 2016/10/14]
3. WHO. Trends in maternal mortality: 1990 to 2015 : estimates by WHO, UNICEF, UNFPA, World Bank Group and the United Nations Population Division. Geneva: World Health Organization, UNICEF, United Nations, Department of Economic and Social Affairs, Population Division, World Bank. , 2015.
4. WHO. WHO Recommendations Intrapartum care for a positive childbirth experience. Geneva: World Health Organization. , 2018.
5. Rowe AK, Rowe SY, Peters DH, et al. Effectiveness of strategies to improve health-care provider practices in low-income and middle-income countries: a systematic review. *Lancet Glob Health* 2018;6(11):e1163-e75. doi: 10.1016/S2214-109X(18)30398-X [published Online First: 2018/10/13]
6. Walker D, Otieno P, Butrick E, et al. Effect of a quality improvement package for intrapartum and immediate newborn care on fresh stillbirth and neonatal mortality among preterm and low-birthweight babies in Kenya and Uganda: a cluster-randomised facility-based trial. *Lancet Glob Health* 2020;8(8):e1061-e70. doi: 10.1016/S2214-109X(20)30232-1 [published Online First: 2020/07/28]
7. Zamboni K, Singh S, Tyagi M, et al. Effect of collaborative quality improvement on stillbirths, neonatal mortality and newborn care practices in hospitals of Telangana and Andhra Pradesh, India: evidence from a quasi-experimental mixed-methods study. *Implement Sci* 2021;16(1):4. doi: 10.1186/s13012-020-01058-z [published Online First: 2021/01/09]
8. Zamboni K, Baker U, Tyagi M, et al. How and under what circumstances do quality improvement collaboratives lead to better outcomes? A systematic review. . *Implement Science* 2020;15(27)
9. Punch K. Introduction to social research. London: SAGE Publications 2005.
10. Siddiqi K, Newell J, Robinson M. Getting evidence into practice: what works in developing countries. *International Journal for Quality in Health Care* 2005;17(5):447-54.
11. Grimshaw JM, Eccles MP, Lavis JN, et al. Knowledge translation of research findings. *Implement Sci* 2012;7:50. doi: 10.1186/1748-5908-7-50 [published Online First: 2012/06/02]
12. Moore GF, Audrey S, Barker M, et al. Process evaluation of complex interventions: Medical Research Council guidance. *BMJ* 2015;350:h1258. doi: 10.1136/bmj.h1258 [published Online First: 2015/03/21]
13. Public Health England. . Guidance Process Evaluation. London: Public Health England. , 2018.
14. Marchal B, Kegels G, Van Belle S. Theory and realist methods. In: Emmel N, Greenhalgh J, Manzano A, et al., eds. Doing Realist Research SAGE London: Sage Publications 2018:79-90.
15. Westhorp G. Using complexity-consistent theory for evaluating complex systems. *Evaluation* 2012;18(4):405-20.
16. Marchal B, Van Belle S, De Brouwere V, et al. Studying complex interventions: reflections from the FEMHealth project on evaluating fee exemption policies in West Africa and Morocco. *BMC Health Services Research* 2013;13:469.
17. Pawson R. The Science of Evaluation. A Realist Manifesto. London: Sage 2013.
18. Wong G, Westhorp G, Manzano A, et al. RAMESES II reporting standards for realist evaluations. *BMC medicine* 2016;14(96)
19. Pawson R, Tilley N. Realistic Evaluation. London: Sage 1997.
20. Marchal B, Van Belle S, van Olmen J, et al. Is realist evaluation keeping its promise? A literature review of methodological practice in health systems research. *Evaluation* 2012;18(2):192-212.

For peer review only

21. Kitson A, Harvey G, McCormack B. Enabling the implementation of evidence based practice: a conceptual framework. *Qual Health Care* 1998;7(3):149-58. doi: 10.1136/qshc.7.3.149 [published Online First: 1998/08/05]
22. Yin R. Case Study Research. Design and Methods. 5 ed. Los Angeles: SAGE 2014.
23. Mukumbang F, Van Belle S, Marchal B, et al. Unravelling how and why the Antiretroviral Adherence Club Intervention works (or not) in a public health facility: A realist explanatory theory-building case study. *Plos One* 2019;14(1):e0210565.
24. Gilmore B. Realist evaluations in low- and middle-income countries: reflections and recommendations from the experiences of a foreign researcher. *BMJ Glob Health* 2019;4(5):e001638. doi: 10.1136/bmjgh-2019-001638 [published Online First: 2019/11/22]
25. Spencer L, Ritchie J, O'Connor W. Carrying out Qualitative Analysis. ; 2003. p. In: Ritchie J, Lewis J, eds. Qualitative research practice: a guide for social science students and researchers. London: SAGE Publications 2003:219–62.
26. WMA. WMA Declaration of Helsinki – Ethical Principles for Medical Research Involving Human Subjects: World Medical Association, 2018.
27. Rogers P. Theory-based evaluation: reflections ten years on. *New Directions for Evaluation* 2007;114(Summer):63-84.
28. Chen H, Rossi P. Evaluating with sense - The theory driven approach. *Evaluation Review* 1983;7:283-302.
29. Connell JP, Kubisch A, Schorr L, et al. New approaches to evaluating community initiatives. Concepts, methods, and contexts. Washington D.C.: The Aspen Institute 1995.
30. Moore G, Audrey S, Barker M, et al. Process evaluation of complex interventions. UK Medical research Council (MRC) guidance: Medical Research Council 2015.
31. Kislov R, Pope C, Martin GP, et al. Harnessing the power of theorising in implementation science. *Implement Sci* 2019;14(1):103. doi: 10.1186/s13012-019-0957-4 [published Online First: 2019/12/12]
32. Merton RK. Social theory and social structure. New York: The Free Press 1968.
33. McIntyre S, Francis J, Gould N, et al. The use of theory in process evaluations conducted alongside randomized trials of implementation interventions: A systematic review. *Translational Behavioral Medicine* 2020;10:168-78.
34. Fletcher A, Jamal F, Moore G, et al. Realist complex intervention science: Applying realist principles across all phases of the Medical Research Council framework for developing and evaluating complex interventions. *Evaluation (Lond)* 2016;22(3):286-303. doi: 10.1177/1356389016652743 [published Online First: 2016/08/02]
35. Rycroft-Malone J, Seers K, Eldh AC, et al. A realist process evaluation within the Facilitating Implementation of Research Evidence (FIRE) cluster randomised controlled international trial: an exemplar. *Implement Sci* 2018;13(1):138. doi: 10.1186/s13012-018-0811-0 [published Online First: 2018/11/18]
36. Pawson R. The science of evaluation: a realist manifesto. London: SAGE Publications 2013.

For peer review only

Word count

4780 words.

Authors' contributions

IOA and BM designed the study, wrote the study protocol, designed the data collection guides and the analytical strategy, drafted the initial manuscript and drafted the final version. VCP, LB, CH and JPD contributed to the study design and the design of the data collection guides. IOA, BM, VCP, LB, CH, JPD, CH, CBM, SM, DM, GN and KSA contributed to the initial draft of the manuscript and to the final version.

Availability of data and materials

Data sharing is not applicable to this article as no datasets were generated or analysed during the current phase of the study.

Funding statement

This study is part of the ALERT project which is funded by the European Commission's Horizon 2020 (No 847824) under a call for Implementation research for maternal and child health. The funder had no role in the design of the study and collection, analysis, and interpretation of data and in writing the manuscript. The contents of this article are solely the responsibility of the authors and do not reflect the views of the European Union.

Competing interests

The authors declare that they have no competing interests.

For peer review only

For peer review only

Acknowledgements

We would like to acknowledge the support of the ALERT study team, which is a consortium of researchers and implementers of eight institutions across Europe, Benin, Malawi, Tanzania and Uganda. This group developed the ALERT project and is responsible for the implementation and the evaluation of the multifaceted intervention. The composition of the group is as follows.

The ALERT Study Team

Ahossi Angèle Florence Laure, Andrea B. Pembe, Ann-Beth Nygaard Moller, Antoinette Sognonvi, Armelle Vigan, Banougnin Bolade Hamed, Beatrice Mwilike, Kéfilath Bello, Bianca Kandeya, Christelle Boyi Metogni, Bruno Marchal, Claudia Hanson, Dickson Mkoka, Effie Chipeta, Elizabeth Ombeva Ayebare, Fadhlun M Alwy Al-Beity, Gertrude Namazzi, Gisele Houngbo, Gottfried Agballa, Hashim Hounkpatin, Helga Naburi, Helle Mölsted Alvesson, Hussein L. Kidanto, Jean-Paul Dossou, Joanne Welsh, Joseph Akuze, Josephine Babirye, Kristi Sidney Annerstedt, Lenka Benova, Lilian Mselle, Mechthild Gross, Muzdalifat Abeid, Nicola Orsini, Peter Waiswa, Philip Wanduru, Razak Mussa, Regine Unkels, Rian Snijders, Samuel Meja, Schadrac Agbla, Therese Delvaux, Tumbwene Mwansisya, Virginia Castellano Pleguezuelo, William Stones, Wim Van Damme, Yesaya Z. Nyirenda, Zamoyoni Julius

Data and Safety Monitoring Board (DSMB)

Jennifer Hall, Erik Lampa, Zahida Qureshi

For peer review only

Figure 1 - The ALERT intervention

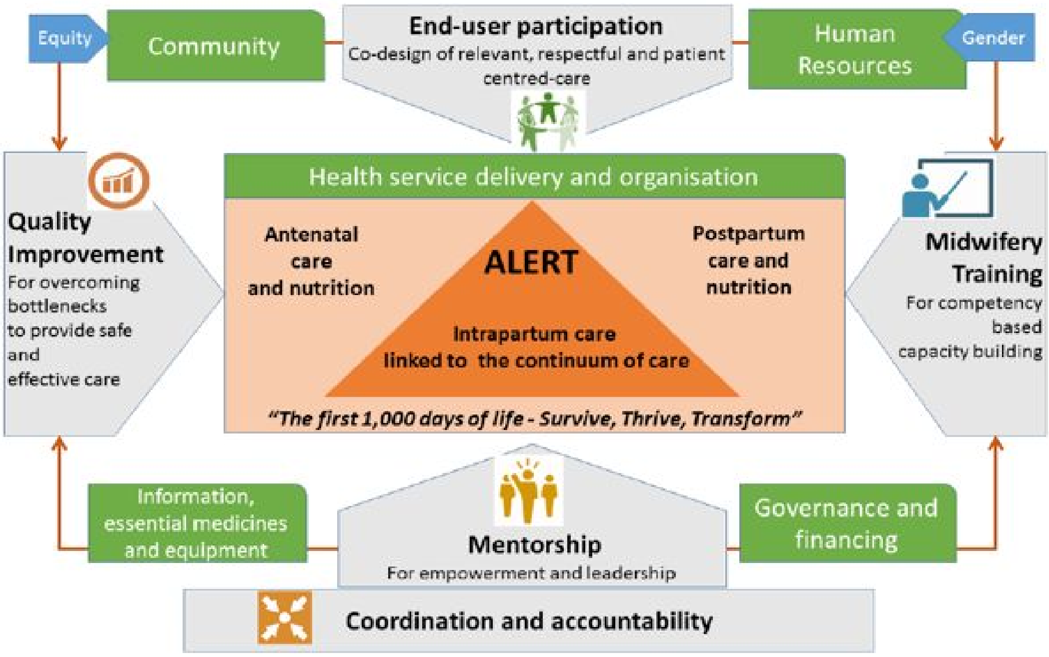

Figure 2 - Sequential integration of hospitals into the trial

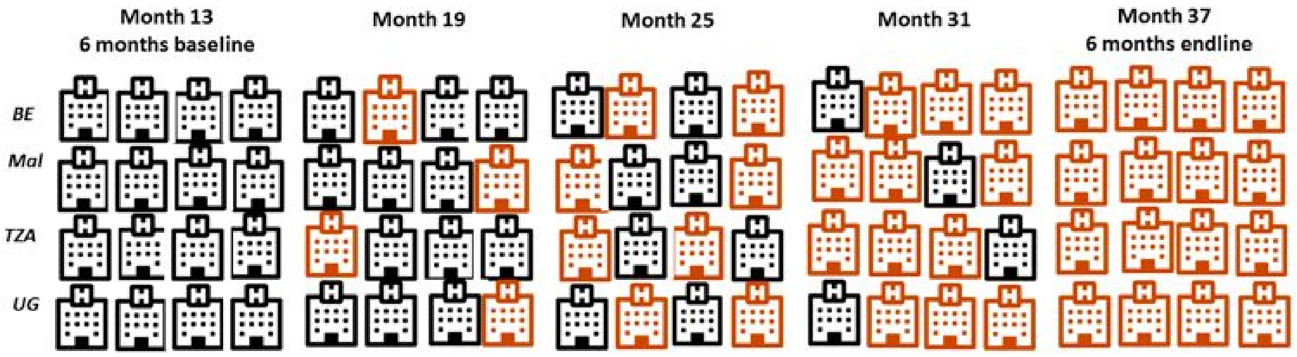

Legend: Pre-intervention (comparison) facilities: black, intervention facilities: red. The indicated months are ALERT program months

For peer review only

Figure 3 – The realist research cycle (source: 20)

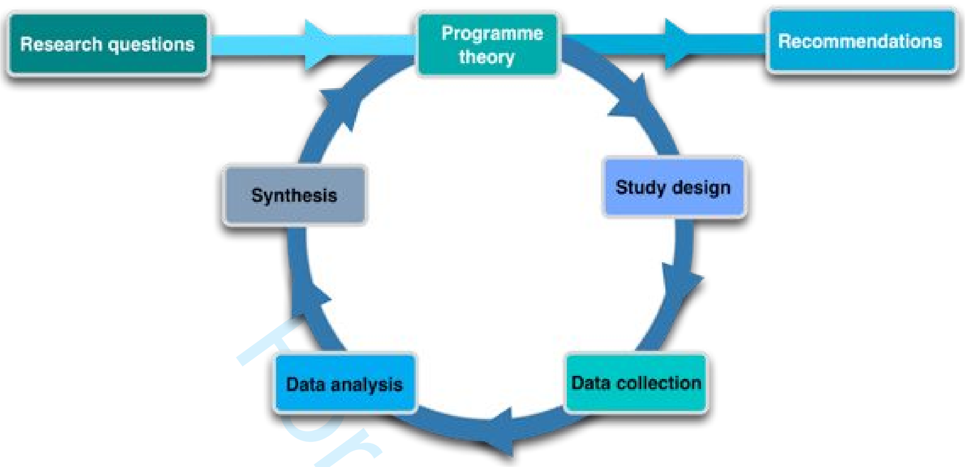

For peer review only

Figure 4 – The ALERT theory of change

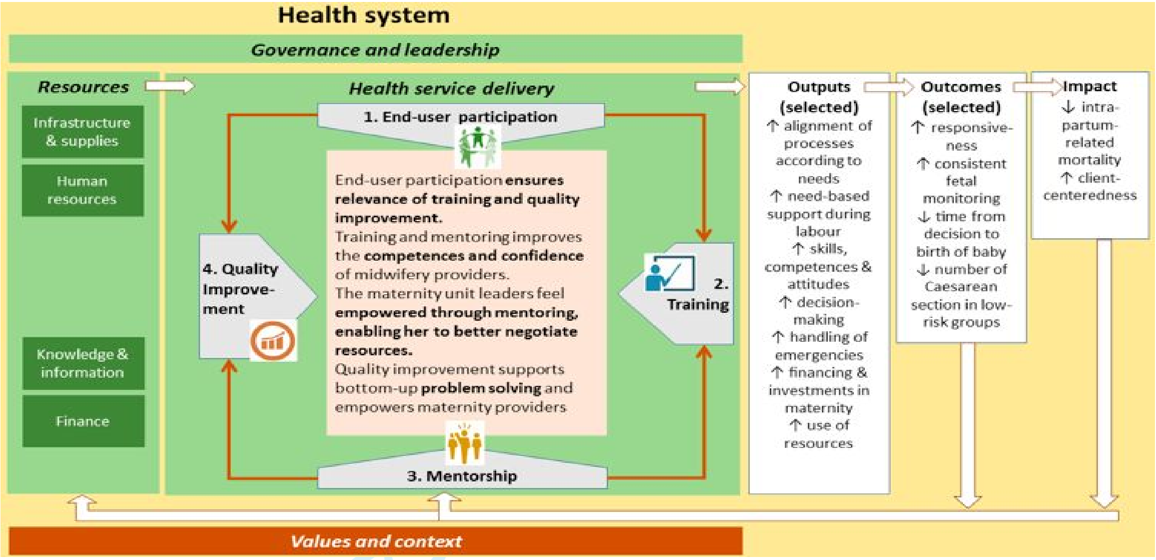

For peer review only

Additional File 1

Characteristics of the study countries

	Benin	Malawi	Tanzania	Uganda
Country-level indicators				
Estimated population (in 2020, million)	12.1	20.3	62.8	47.2
Maternal mortality ratio per 100,000 live births (2015) [3]	405	634	398	343
Neonatal mortality rate per 1,000 live births (2018) [1]	32.7	22.7	21.1	20.2
Stillbirth rate per 1,000 total births (2015) [102]	30.3	21.8	22.4	21.0
% of births in health facilities (DHS Statcompiler) #	86.9%	91.4%	62.6%	73.4%
Annual growth rate in % of births in health facilities #	1.3%	3.2%	2.6%	4.4%
% of facility births in hospitals #	55.1%	42.2%	47.8%	47.8%
% of all live births by caesarean section # / Poorest v Richest wealth quintile	5.4%<br>2.7% - 11.5%	6.1%<br>3.0% - 9.1%	5.9%<br>2.4% - 15.8%	6.2%<br>2.7% - 14.2%
Among live births in health facilities				
% of women checked by a health professional before discharge from facility following childbirth #	68%	57%	51%	47%
% of all live births by caesarean section #	6.3%	6.3%	9.5%	8.3%
Among live births in hospitals				
Neonatal mortality per 1,000 live births #	20.6	35.8	31.8	27.1
% of newborns breastfed within 1 hour of birth #	67.8%	73.1%	54.9%	65.3%
Health system indicators				
Doctors /10,000 people population ^	1.6 (2016)	1.2 (2016)	0.4 (2014)	0.9 (2015)
Nursing cadres /10,000 people population ^	6.2 (2016)	2.5 (2016)	4.1 (2014)	6.3 (2015)
Predominant midwifery provider [103]	Midwife	Nurse-midwife	Nurse-midwife	Midwife
Hospital beds / 10,000 people population ^	5 (2010)	13 (2011)	7 (2010)	5 (2010)
Current health expenditure per capita (USD PPP, 2015) ^	85	108	97	139
Out-of-pocket expenditure as % of current health expenditure (2015?)^	41	11	26	41
User fees for childbirth (vaginal/caesarean) ^	Official fees	No official	No official	No official

additional analysis of Demographic and Health Survey data, Benin; 2011-12; Malawi: 2015-16; Tanzania: 2015-16; Uganda: 2016
^WHO observer http://apps.who.int/gho/data/node.main.HWFGRP_0020?lang=en

For peer review only

For peer review only

Additional File 2
Checklist for Realist evaluation studies

Based on Wong, G., G. Westhorp, A. Manzano, J. Greenhalgh, J. Jagosh and T. Greenhalgh (2016). "RAMESES II reporting standards for realist evaluations." BMC Medicine **14**(96).

		Page	Comment
TITLE	In the title, identify the document as a realist evaluation	Done on P1.	
SUMMARY OR ABSTRACT	Journal articles will usually require an abstract, while reports and other forms of publication will usually benefit from a short summary. The abstract or summary should include brief details on: the policy, programme or initiative under evaluation; programme setting; purpose of the evaluation; evaluation question(s) and/or objective(s); evaluation strategy; data collection, documentation and analysis methods; key findings and conclusions Where journals require it and the nature of the study is appropriate, brief details of respondents to the evaluation and recruitment and sampling processes may also be included Sufficient detail should be provided to identify that a realist approach was used and that realist programme theory was developed and/or refined		We followed the guidance published by Wong, which was originally developed for guiding the reporting of finished realist evaluation studies, and adapted it to fit a protocol paper. As a consequence, not all the required information is located in the sections proposed by the Guidance.
INTRODUCTION			
3 Rationale for evaluation	Explain the purpose of the evaluation and the implications for its focus and design	This is presented on P5 at the start of the Methods section.	
4 Programme theory	Describe the initial programme theory (or theories) that underpin the programme, policy or initiative	The Theory of change of the intervention, which will be the starting point of the development of the initial programme theory is presented on P7.	

For peer review only - http://bmjopen.bmj.com/site/about/guidelines.xhtml

5 Evaluation questions, objectives and focus	State the evaluation question(s) and specify the objectives for the evaluation. Describe whether and how the programme theory was used to define the scope and focus of the evaluation	These are presented on P6-7.	
6 Ethical approval	State whether the realist evaluation required and has gained ethical approval from the relevant authorities, providing details as appropriate. If ethical approval was deemed unnecessary, explain why	The list of approvals of the ethical review boards is presented on P5.	
METHODS			
7 Rationale for using realist evaluation	Explain why a realist evaluation approach was chosen and (if relevant) adapted	Presented on P5 in the opening paragraphs of section Methods.	
8 Environment surrounding the evaluation	Describe the environment in which the evaluation took place	A table with the key characteristics of the four study countries is presented in Additional file 1.	
9 Describe the programme policy, initiative or product evaluated	Provide relevant details on the programme, policy or initiative evaluated	Presented in the Introduction on p. 4-5	
10 Describe and justify the evaluation design	A description and justification of the evaluation design (i.e. the account of what was planned, done and why) should be included, at least in summary form or as an appendix, in the document which presents the main findings. If this is not done, the omission should be justified and a reference or link to the evaluation design given. It may also be useful to publish or make freely available (e.g. online on a website) any original evaluation design document or protocol, where they exist.	This is extensively presented on P5-13	
11 Data collection methods	Describe and justify the data collection methods – which ones were used, why and how they fed into developing, supporting,	For each specific objective, we present the data collection methods (P8, 9, 10). On P12, we present an	

For peer review only - http://bmjopen.bmj.com/site/about/guidelines.xhtml

	refuting or refining programme theory<br>Provide details of the steps taken to enhance the trustworthiness of data collection and documentation	overview in the form of a table.	
12 Recruitment process and sampling strategy	Describe how respondents to the evaluation were recruited or engaged and how the sample contributed to the development, support, refutation or refinement of programme theory	This is presented on P9.	
13 Data analysis	Describe in detail how data were analysed. This section should<br>include information on the constructs that were identified, the<br>process of analysis, how the programme theory was further<br>developed, supported, refuted and refined, and (where relevant)<br>how analysis changed as the evaluation unfolded	For specific objective 2 and 3, we present the data analysis strategy methods (P10 and 11).	
RESULTS			
14 Details of participants	Report (if applicable) who took part in the evaluation, the<br>details of the data they provided and how the data was used<br>to develop, support, refute or refine programme theory	N/A	
15 Main findings	Present the key findings, linking them to contexts, mechanisms<br>and outcome configurations. Show how they were used to<br>further develop, test or refine the programme theory	N/A	
DISCUSSION			
16 Summary of findings	Summarise the main findings with attention to the evaluation<br>questions, purpose of the evaluation, programme theory and<br>intended audience	N/A	

For peer review only - http://bmjopen.bmj.com/site/about/guidelines.xhtml

17 Strengths, limitations and future directions	Discuss both the strengths of the evaluation and its limitations.<br>These should include (but need not be limited to):<br>(1) consideration of all the steps in the evaluation processes; and (2) comment on the adequacy, trustworthiness and value of the explanatory insights which emerged<br>In many evaluations, there will be an expectation to provide guidance on future directions for the programme, policy or initiative, its implementation and/or design. The particular implications arising from the realist nature of the findings should be reflected in these discussions	Presented in Box Strengths and limitations (P2)	
18 Comparison with existing literature	Where appropriate, compare and contrast the evaluation's findings with the existing literature on similar programmes, policies or initiatives	N/A	
19 Conclusion and recommendations	List the main conclusions that are justified by the analyses of the data. If appropriate, offer recommendations consistent with a realist approach	N/A	
20 Funding and conflict of interest	State the funding source (if any) for the evaluation, the role played by the funder (if any) and any conflicts of interests of the evaluators	Presented on P2 and in the a-Acknowledgements	

For peer review only - http://bmjopen.bmj.com/site/about/guidelines.xhtml

BMJ Open

Strengthening capacity in hospitals to reduce perinatal morbidity and mortality through a co-designed intervention package: protocol for a realist evaluation as part of a stepped-wedge trial of the Action Leveraging Evidence to Reduce perinatal morTality and morbidity in sub-Saharan Africa (ALERT) project

Journal:	*BMJ Open*
Manuscript ID	bmjopen-2021-057414.R1
Article Type:	Protocol
Date Submitted by the Author:	15-Mar-2022
Complete List of Authors:	Abejirinde, Ibukun-Oluwa; Hospital for Sick Children, International Program Evaluation Unit, Centre for Global Child Health ; University of Toronto Dalla Lana School of Public Health, Division of Social & Behavioural Health Sciences<br>Castellano Pleguezuelo, Virginia; Institute of Tropical Medicine<br>Benova, Lenka; Institute of Tropical Medicine, Department of Public Health<br>Dossou, Jean-Paul; Centre de Recherche en Reproduction Humaine et en Démographie<br>Hanson, Claudia; Karolinska Institute, Dept Public Global Health<br>Metogni, Christelle Boyi; Centre de Recherche en Reproduction Humaine et en Démographie<br>Meja, Samuel; University of Malawi College of Medicine<br>Mkoka, D; Muhimbili University of Health and Allied Sciences<br>Namazzi, Gertrude; Makerere University, Department of Health Policy Planning and Management<br>Sidney, Kristi; Karolinska Institute, Global Public Health<br>Marchal, Bruno; Institute of Tropical Medicine, Department of Public Health
**Primary Subject Heading**:	Obstetrics and gynaecology
Secondary Subject Heading:	Health services research, Health policy, Nursing, Paediatrics, Research methods
Keywords:	EDUCATION & TRAINING (see Medical Education & Training), Clinical governance < HEALTH SERVICES ADMINISTRATION & MANAGEMENT, Quality in health care < HEALTH SERVICES ADMINISTRATION & MANAGEMENT, OBSTETRICS, PERINATOLOGY

SCHOLARONE™
Manuscripts

Strengthening capacity in hospitals to reduce perinatal morbidity and mortality through a co-designed intervention package: protocol for a realist evaluation as part of a stepped-wedge trial of the Action Leveraging Evidence to Reduce perinatal morTality and morbidity in sub-Saharan Africa (ALERT) project

Ibukun-Oluwa Omolade Abejirinde[1], Virginia Castellano Pleguezuelo[2], Lenka Benova[2], Jean-Paul Dossou[3], Claudia Hanson[4,5], Christelle Boyi Metogni[3], Samuel Meja[6], Dickson Mkoka[7], Gertrude Namazzi[8], Kristi Sidney Annerstedt[4] and Bruno Marchal[2]

Affiliations

[1] Social and Behavioural Health Sciences Division, Dalla Lana School of Public Health, University of Toronto, Toronto (Canada)

[2] Department of Public Health, Institute of Tropical Medicine, Antwerp (Belgium)

[3] Centre de Recherche en Reproduction Humaine et en Démographie (CERRHUD), Cotonou, Benin

[4] Department of Global Public Health, Karolinska Institutet, Sweden

[5] Department of Disease Control, London School of Hygiene and Tropical Medicine, London, United Kingdom

[6] College of Medicine, The Centre for Reproductive Health, University of Malawi, Blantyre, Malawi

[7] Department of Obstetrics and Gynaecology, Muhimbili University of Health and Allied Sciences, Dar Es Salaam, Tanzania

[8] Centre of Excellence for Maternal Newborn and Child Health, Department of Health Policy Planning and Management, School of Public Health, Makerere University, Uganda

For peer review only

Corresponding author

Bruno Marchal, Department of Public Health, Institute of Tropical Medicine, Antwerp

Nationalestraat 155, 2000 Antwerp, Belgium

bmarchal@itg.be

+32.2476384

Abstract

Introduction

Despite a strong evidence base for developing interventions to reduce child mortality and morbidity related to pregnancy and delivery, major knowledge-implementation gaps remain. The Action Leveraging Evidence to Reduce perinatal morTality and morbidity in sub-Saharan Africa (ALERT) project aims to overcome these gaps through strengthening the capacity of multidisciplinary teams that provide maternity care. The intervention includes competency-based midwife training, community engagement for study design, mentoring, and Quality Improvement cycles. The realist process evaluation of ALERT aims at identifying and testing the causal pathway through which the intervention achieves its impact.

Methods and analysis

This realist process evaluation complements the effectiveness evaluation and the economic evaluation of the ALERT intervention. Following the realist evaluation cycle, we will first elicit the initial programme theory on the basis of the ALERT theory of change, a review of the evidence on adoption and diffusion of innovations, and the perspectives of the stakeholders. Second, we will use a multiple embedded case study design to empirically test the initial programme theory in two hospitals in each of the four intervention countries. Qualitative and quantitative data will be collected, using in-depth interviews with hospital staff and mothers, observations, patient exit interviews and (hospital)

For peer review only

document reviews. Analysis will be guided by the Intervention-Actors-Context-Mechanism-Outcome configuration heuristic. We will use thematic coding to analyse the qualitative data. The quantitative data will be analysed descriptively and integrated in the analysis using a retroductive approach. Each case study will end with a refined programme theory (in-case analysis). Third, we will carry out a cross-case comparison within and between the four countries. Comparison between study countries should enable identifying relevant context factors that influence effectiveness and implementation, leading to a mid-range theory that may inform the scaling up the intervention.

Ethics and dissemination

In developing this protocol, we paid specific attention to cultural sensitivity, the *do no harm* principle, confidentiality and non-attribution. We received ethical approval from the local and national institutional review boards in Tanzania, Uganda, Malawi, Benin, Sweden and Belgium. Written or verbal consent of respondents will be secured after explaining the purpose, potential benefits and potential harms of the study using an information sheet. The results will be disseminated through workshops with the hospital staff and national policymakers, and scientific publications and conferences.

Keywords

Perinatal mortality, capacity development, quality improvement, process evaluation, realist evaluation

Strengths and limitations of this study

- Realist evaluation enhances the explanatory power of process evaluations by providing an analytical approach that starts from an intervention theory and explains the outcome in terms of a configuration of intervention, context, actors and mechanisms of change.

For peer review only

- The approach furthermore allows testing and developing the theoretical basis of implementation and scaling up of effective interventions.
- Study limitations include social desirability bias and potential risks presented by cross-cultural differences, power imbalances and western-dominated theories in realist research.

Introduction

Child mortality and mortality related to pregnancy and childbirth remain major public health priorities. Globally, five million children die before their second birthday [1] and nearly 7.5 million children die within the first 1000 days of life. Over two million babies are stillborn each year[2] and for almost 300,000 women annually, pregnancy or childbirth are fatal.[3] While there is strong evidence available related to what ought to be made available to women and their new-borns during childbirth[4], there is a lack of evidence on how best to support health providers to implement the evidence-based practices which can save lives and prevent suffering. The Action Leveraging Evidence to Reduce perinatal morTality and morbidity in sub-Saharan Africa (ALERT) project was developed to close the gap between evidence and practice. ALERT is a 5-year multifaceted health system intervention targeting the intrapartum period. It is funded by the European Commission under the Horizon 2020 framework and led by Karolinska Institute in collaboration with seven other institutions. National universities and health care professional organisations in cooperation with local district health management teams and local training institutions will deliver the ALERT intervention.

The project aims to (1) overcome the knowledge-implementation gap at the level of multidisciplinary teams providing maternity care and (2) promote implementation of evidence-based interventions in hospitals with a target primary outcome of reduced in-facility perinatal (stillbirths and early neonatal) mortality. The ALERT project will develop an intervention that includes four main components (Figure 1):

For peer review only

1. Co-design of the intervention via end-user participation, involving narratives of women, families and midwifery providers. These include nurses, nurse-midwives, midwives, auxiliary staff, physicians and obstetricians.

2. Competency-based training for midwives

3. Quality improvement cycles to help providers identify and overcome problems, supported by data from a clinical perinatal e-registry

4. Mentoring maternity unit leaders to foster empowerment and leadership, complemented by bi-annual coordination and accountability meetings at district level.

The intervention is based on a framework that builds on many years of experience working in the respective settings and the literature on quality improvement and learning (including, for instance, the work of Rowe *et al*.[5] and Walker *et al.*[6] It was deliberately designed to integrate the above-described four components to support health care workers in providing safe and respectful intrapartum care. This combination is expected to leverage previous experience suggesting that in the ALERT settings, knowledge deficits remain an important bottleneck and that this may create better engagement into QI.[6,7] The QI component will build on the work and processes put in place by existing quality improvement teams and death review committees. Their inputs will guide the identification of the topics to be covered by the QI component, which will be aligned to the training for the midwifery care providers and use the Do-Study-Act (PDSA) cycle. Mentoring by valued mentors, an essential aspect of QI[8], will be carefully designed and include a cascade approach to meet the needs of the health care workers. While the ALERT project is based on global evidence, the intervention will, indeed, be adapted to the context and needs of each hospital, integrating the perspectives of midwifery providers as well as women and their families. The intervention was field tested during a visit to a regional hospital in Dar-es-Salaam, Tanzania in February 2019.

For peer review only

The implementation started in Benin, Malawi, Tanzania and Uganda in 2020 and will run until 2024, steered by seven scientific work packages (WPs). Four district-level hospitals were selected in each country, representing a mix of public and private not-for-profit facilities. Each hospital has at least 2.500 births per year and this selection should provide sufficient power (75–80%) to detect a 25% reduction in early perinatal mortality with 95% confidence intervals. We refer to the overall study protocol paper for more details.[9] The intervention will be rolled out as indicated in Figure 2. The hospitals were randomly selected for inclusion. More background information for each country is provided in Additional file 1.

An implementation science approach is at the heart of this study. Since the implementation of evidence-based guidelines (and arguably any quality improvement strategy) is not only determined by providers, funding and health care system capacity[10], but also by its adoption and implementation by individuals and teams, it is critical to understand what works and why.[11,12] Three approaches will be employed to evaluate the ALERT intervention:

i. A stepped-wedge design to evaluate the effect of the intervention on in-facility perinatal mortality with a primary outcome of reduced in-facility perinatal (stillbirths and early neonatal) mortality

ii. A realist process evaluation to understand what works, for whom, and under which conditions, complemented by an economic evaluation

iii. Economic evaluation focused on scalability including costing studies in the countries.

In this paper, we present the research protocol of the realist process evaluation, which is part of a stepped wedge trial. Process evaluations (PE) are often carried out in implementation research, not only to address the issue of the black box between an intervention and its results, but also because they are considered as useful in the design and testing of complex interventions.[13] A process evaluation considers the actual implementation of the programme, the underlying mechanisms of

For peer review only

change and the effect of context on outcomes.[14] To this end, process evaluations often incorporate an assessment of implementation (fidelity), a stakeholder analysis, and a context mapping. We developed the process evaluation on the basis of realist evaluation because of its explanatory potential: realists assume that interventions lead to effects in specific contexts, by triggering mechanisms of change for specific groups of actors. This configurational approach to causation allows for greater in-depth understanding of why and for whom intervention work (or not).[15] Over the years, RE has become recognized in health policy and systems research as a useful methodology for evaluating complex interventions.[16,17,18] This study contributes to filling a gap, as few studies in the field of implementation science have adopted the realist evaluation approach. We followed the RAMESES II guidance for reporting on realist evaluations[19], which we have adapted to fit the format for a protocol paper (see Additional file 2).

Methods and analysis

The overall methodological approach

In the quest to open the 'black-box' of complex programmes, Pawson and Tilley developed realist evaluation (RE), a theory-driven research approach that seeks to understand the links between context, outcomes and mechanisms.[20] While traditional evaluation questions often focus on effectiveness or on process, RE seeks to answer a string of questions: *what works, for whom, in what respect, to what extent, in what context and how?* From the perspective of RE, programmes work (or not) because actors make decisions in response to the opportunities or resources that the programme provides. According to Pawson and Tilley, programmes or interventions can be viewed as theories or assumptions held by people (including designers, implementers, beneficiaries, and policymakers) who are embedded in the open social systems in which they operate. Therefore, it is not the programme, but the actors who are engaged in it who bring about the results. To understand how that happens, RE explores the context in which the programme operates and the mechanisms that are underlying

For peer review only

the observed change (or the lack thereof). Mechanisms refer to psychological, social, cultural or organisational factors that explain why actors respond to a programme the way they do. This response, however, does not happen in isolation; the context in which the programme takes place influences the way actors respond to it: mechanisms (and thus the programmes that trigger them) only work if the context conditions are right. Summarily, not every programme works identically in different settings. Realist evaluators use the term *programme theory (PT)* to describe the explanation of how a programme has contributed to observed results. The programme theory is not only the end product of a realist evaluation, but it is also a hypothesis that serves as the starting point of any realist evaluation. The initial PT shapes the study design, data collection, analysis and synthesis.

We structured the process evaluation of ALERT according to the steps of the realist research cycle.[21]

- In consideration of the topic being researched, realists build the **initial PT** upon existing evidence (eg. literature reviews, document reviews), interviews with key actors and/or exploratory research.
- The **study design** is chosen as a function of the programme theory that needs to be assessed.
- **Data collection** tools and methods are selected based on the components of the initial programme theory to be tested: all data required to test the initial PT should be collected. Often mixed methods (qualitative and quantitative) of data collection are used.
- **Data analysis**: the Intervention-Context-Actor-Mechanism-Outcome (ICAMO) configuration heuristic is often used in the analysis of data. [15] Qualitative data analysis is usually based on a thematic coding approach using the core elements of the initial PT. The quantitative data are often analysed with descriptive statistics and integrated in the analysis using a retroductive approach.
- Each study ends with a **synthesis** based on a comparison of the findings of the study with the initial PT, which is adapted as needed, leading to a refined PT.

For peer review only

Objectives

The main objective of the ALERT process evaluation is to identify and test the causal pathway through which the intervention achieves its impact. Summarily, it aims to answer the question *"Which components of the ALERT intervention work, how, for whom and in which contexts?"*

There are four specific objectives:

i. To collaboratively elicit the initial programme theory of the ALERT intervention

ii. To test the initial programme theory through a multiple embedded case study

iii. To refine the programme theory of the ALERT intervention using cross-case analysis

iv. To summarise and disseminate findings in the form of recommendations to inform implementation and practice (of quality intrapartum care) at hospital and country levels.

In the following sections, each of the four core objectives and the corresponding data collection and analysis methods are presented.

Objective 1: Defining the Initial Programme Theory

We will formulate the initial ALERT PT based on its Theory of Change (TOC) (see Figure 3), a review of the existing evidence on adoption and diffusion of innovations, and the perspectives of stakeholders involved in the intervention.

ALERT's theory of change is informed by the integrated Promoting Action on Research Implementation in Health Services (i-PARIHS) framework.[22] It identifies the *what* (i.e. an intervention that fits with existing practice, is user-friendly, has a comparative advantage and can be tested); the *who* or *recipients* (i.e. end users: midwifery providers, mothers and their families, who are motivated and

For peer review only

have sufficient skills and resource support by collaboration and teamwork); the *where* or *context* (sub-Saharan African (SSA) hospitals with formal and informal leadership support, effective evaluation and feedback processes and a conducive learning environment) and the *how* (i.e. a problem-focused multi-faceted approach to ensure participation and involvement, ownership and control, and empowerment within and between groups). The TOC implies that end-user participation ensures that the needs of women, families and midwifery providers (ie. the end users) are identified and addressed in the form of training and quality improvement (QI). Also, better trained and supported midwifery providers will provide better care, including improved foetal monitoring, improved emergency care, and client-centred support during labour. Furthermore, better skilled and empowered midwifery providers will also be better able to quickly make decisions. Finally, improved hospital processes leading to timely access to caesarean section or operative vaginal delivery will reduce hypoxic-ischaemic events and thus reduce stillbirths and neonatal deaths.

In order to move the TOC to the level of an initial programme theory, we would need to identify the context elements and mechanisms that would explain how and why the ALERT intervention would achieve the expected results. We plan to carry out the following steps:

- We will summarise existing evidence to identify any theories that may explain how and in which conditions the ALERT intervention will be effective. More concretely, we will focus on the adoption, diffusion, and maintenance of the intervention (components) by health care providers and hospital managers, and the role of multi-stakeholder co-production in these processes.
- We will draw upon health facility assessment data, stakeholder mapping data and the national context assessment data collected by other work packages in the intervention, to describe the national and facility context, and the views and perspectives of national stakeholders.
- We will organise participatory stakeholder workshops to elicit the assumptions of the ALERT consortium members.

For peer review only

- We will use the opportunity of online and face-to-face monthly coordination meetings, consortia meetings, discussions and feedback sessions to present and discuss the evolving initial programme theory to our colleague-researchers and stakeholders from each country.

The end product of these is the initial programme theory that will guide the further steps of the process evaluation.

Objective 2: A multiple embedded case study to test the initial PT

To test the initial PT, we will use a case study design. Investigating the intervention's implementation through case studies in hospitals in four countries will allow us to monitor the actual implementation of the intervention and how it progressively evolves (in terms of actual implementation, context conditions, response of actors and potential mechanisms) across the study sites.

The case study design

We will carry out a multiple embedded case study.[23] We define 'the case' as the implementation of the ALERT intervention. We define 'study site' as a hospital implementing the ALERT intervention. In realist evaluation, study sites are chosen purposively in order to allow testing the initial PT (ie. theoretical sampling). In practice, in each country, we will select two of the four study sites, whereby we aim to choose contrasting sites that are likely to 'manifest' the IPT differently due to contextual variation.

In each country, the first site selected for the realist process evaluation is the hospital where the ALERT intervention will start at step 1 (Month 19 [M19] - M24) of the stepped wedge intervention design (Figure 2). In these four hospitals, we will conduct a detailed "thick" evaluation (see details on data collection below). The second hospitals will be chosen among the stage 3 hospitals (M31 - M36 of implementation), and a "light" evaluation will be conducted. The phased recruitment of hospitals into the realist process evaluation will enable the assessment of how length of exposure to the intervention and changes over time influence outcomes. Selecting two hospitals per country will allows us to

For peer review only

conduct in-country cross-case comparisons and to identify how mechanisms play out differently at country and facility levels.

Data collection

We will use the following data collection methods and tools:

- In each site, we will carry out in-depth interviews with health stakeholders (including midwives, maternity unit managers, hospital managers, the district director of health), focusing on their view on the reasons the ALERT intervention works (or not).
- This will be complemented by opportunistic observations and review of relevant hospital documents.
- We will draw upon data collected by WP3 (training module development), WP4 (health facility assessment), WP5 (perinatal data) and WP 6 (evaluation and fidelity monitoring) to describe the planned intervention (end product of WP3), the actual implementation of the intervention (WP6), as well as the context and the actors (WP4) and the processes triggered by the intervention (WP6). For more details about these work packages, we refer to the overall protocol paper.[9]
- We will carry out in-depth interviews with mothers and their families to document their views on the design and implementation of the ALERT intervention.
- We will draw upon the data from patient exit interviews carried out by another ALERT work package to obtain additional information on the views of patients on the care they received and the (potential) influence of the ALERT intervention.

Intervals between data collection offer time to analyse data and refine the IPT based on emerging patterns which will be presented at the subsequent consortium meetings. Table 1 summarises the data collection plan.

Table 1. Summary of ALERT Realist process evaluation data collection

For peer review only

Specific objective	Data collection methods	Product/finality
Defining the Initial Programme Theory	i. Scoping review<br>ii. ALERT Consortium stakeholder workshop<br>Iii. Country level stakeholder workshops (x4)<br><br>Data used from other work packages<br>i. Health facility assessments and Bottleneck analysis<br>ii. Implementation process description	The initial programme theory of the ALERT intervention
Multiple embedded case study to test the IPT	i. Context mapping (hospital)<br>ii. Context mapping (national/health system)<br>iii. In-depth interviews with women and their families<br>iv. In-depth interviews with midwives, maternity unit managers, hospital managers, district directors of health<br><br>Data used from other work packages<br>i. Review data collection by WP 2 and 3 from narratives and ethnographic observations of women and health providers	Site-specific ICAMO configurations and potentially programme theories
Cross-case analysis to refine the programme theory of ALERT	This step involves first the comparison of the country-specific programme theories in order to identify patterns of similarities and differences.<br>In a second step, the findings are brought to a next level of abstraction, aimed at capturing the essential elements that explain the results of each site in a comprehensive manner	i. Country-specific programme theories<br>ii. Overarching programme theory

Data collection tools

For peer review only

For each data collection method described above, specific tools will be used: a facilitator guide for the participatory stakeholder workshops for ALERT consortium members; in-depth interview guides for hospital managers/in-charges, midwives and district directors of health; and in-depth interview guides for mothers and families (the latter interviews will be carried out within another work package). Local research assistants will collect data in each country. To bridge gaps in technical capacity, especially with respect to realist interviewing techniques[24], we will conduct face-to-face training, complemented by remote training and support.

Data management

Audio files (and handwritten notes) will be translated, transcribed verbatim, and managed for analysis using NVIVO© qualitative data analysis software. Translation will aim to retain technical and cultural meaning in order to minimise interpretation gaps resulting from cross-cultural differences.[25] A random selection of audio files and their corresponding transcripts will be reviewed and checked for quality and accuracy by a member of the realist process evaluation team. Audio files and interview transcripts will be tagged alpha-numerically based on date, district identifier and category of stakeholder. Individuals will not be identified by their real names in field notes. After each data collection session, all information (raw and transcribed data) will be stored on password-protected devices (external drives, computers) and will only be accessed by evaluation team members.

Data analysis

As mentioned above, RE is method-neutral: discipline-specific and appropriate techniques are adopted during data analysis. In general, the analysis will be guided by the ICAMO configuration. Analysis of the qualitative data will be guided by the primary elements and themes identified in the initial ALERT programme theory. Analysis of transcripts will begin immediately following transcription, allowing for early identification of ICAMO configurations and patterns. In line with realist principles, a

For peer review only

thematic coding approach will be used that is based on the core elements of the initial PT. The framework analysis method [25] suits the realist approach as it allows for the inclusion of both a priori and emergent concepts.

- The data will be categorised in a first round of analysis using the ICAMO configuration.
- New interpretations will emerge in subsequent rounds of coding, leading to a refined analysis.
- Next, a retroduction approach will be adopted, whereby the observed outcomes are explained by looking into the mechanisms, actual intervention modalities, actors and context elements. This results in descriptions of the actual intervention, its effects (both intended and unintended, positive and negative), the context elements and the underlying mechanisms (ICAMO configurations), which offer the most plausible explanations of the observed outcomes.

During this analytical process, we will also draw upon data and findings from other work packages and integrate participatory reflection and feedback from the ALERT consortium annual meetings. Data triangulation and verification will be ensured by comparing responses from different respondents and from other data sources in order to identify similar and divergent views. This will contribute to the internal validity of the analysis.

Synthesis

The site-specific ICAMO configuration(s) will be compared with the initial PT, and in a process of abstraction, the initial PT will be modified when and where needed.

Objective 3: Cross-case analysis to refine the programme theory of ALERT

The above described "in-case" analysis will be followed by cross-case analysis within and between the countries. Comparing between the study countries should enable identifying relevant context factors at country level and assessing their influence on intervention implementation and scale up. In practice, we will develop tables presenting the different ICAMO configurations by country. We will look for patterns of similarity and difference and see how these challenge, refute or confirm the initial

For peer review only

programme theory. Prior to the finalisation of the refined programme theory, the results of the process evaluation will be discussed in a final round of participatory workshops with ALERT partners and in-country stakeholders directly involved in the project. This will provide an opportunity to contextualise, reflect on and improve the programme theory.

In summary, the final round of PT development will occur iteratively during the cross-case analysis, leading to a final programme theory that may reach the level of a mid-range theory, which indicates what it is about the ALERT intervention that works for whom and in which circumstances. The results may not only inform the formulation of recommendations for scaling up the intervention in the study countries, but may also allow tailoring of similar interventions to different contexts.

Objective 4: Dissemination

To summarise and disseminate findings in the form of recommendations at study site and country level, we will leverage the annual consortium project meetings. This also serves as an opportunity for cross-pollination of experiences and learning between intervention countries, and for presenting and discussing the research findings within the broader findings of the project. Feedback meetings will adopt a participatory open discussion approach with the aim to discuss potential and critical action points for improving implementation and scaling up to other locations, as well as the possible implications (positive and negative) of such efforts.

In addition to the final country reports on the RE from each country, findings will be further disseminated through at least two open-access publications and two international conferences. We expect that the doctoral student working on this process evaluation will provide additional avenues for dissemination at country and international level (conferences and peer-reviewed publications).

Patient and public involvement

For peer review only

In this component of the ALERT project, which in essence is an evaluation of a quality improvement intervention, patients nor members of the public were involved in the design and they will not be involved in the actual evaluation and analysis. However, during the formative phase of the project, the ALERT intervention will be co-designed with women and families living in the study sites. Their narratives regarding pregnancy, birth and their health seeking behaviour in general will be documented and analysed in order to inform the intervention design. They will be included in feedback meetings at the end of the project.

Ethics and dissemination

The research team recognizes the ethical implications of any form of research. The realist process evaluation will be carried out in line with the principles of the Declaration of Helsinki as amended in 2013, and any further versions.[26]

General principles

The study team will place particular emphasis on:

- *Cultural sensitivity:* Data collection modalities will be aligned to the socio-cultural norms and preferences of people in their daily life and within organizational settings (for example, ensuring interviews with health workers does not disrupt health service provision and other duties). All personnel on the study team will be bound to the code of conduct and ethical restrictions of the research.
- *Do no harm:* Respondents will be well informed of their rights to withdraw from the study at any point in time and all efforts will be made to ensure that no discomfort or stress is experienced by respondents as a result of the research. This includes allowing respondents to choose their preferred location and time for interviews with care taken to ensure privacy to

For peer review only

the greatest degree possible. Informed consent, privacy and confidentiality, as well as respect for participants' time will be prioritized.

- *Confidentiality and non-attribution:* Only ALERT team members will have access to raw data. All documents, completed questionnaires and audio recordings will be treated with the strictest care for confidentiality. Reports will be compiled with the intention to protect the identity of respondents while representing their views and opinions as accurately and fairly as possible.

Vulnerability of the target populations

The views and experiences of respondents for the realist process evaluation are primarily collected through participatory sessions and in-depth interviews (ie. qualitative methods). We consider that the issues to be discussed in interviews are of a non-sensitive nature and we do not foresee any harmful risks or adverse outcomes (principle of non-maleficence). Due to the nature of health workers' jobs, interviews will be conducted at a place and time of their convenience without hindering their service delivery tasks. Respondents will, however, be making a sacrifice of their time in order to participate in the interviews. They will be compensated in line with the country standards for transportation re-imbursement and provision of refreshments (water, non-alcoholic beverages, and snacks).

Informed Consent

Written or verbal consent of respondents will be secured after explaining the purpose, potential benefits and potential harms of the study using an information sheet. Copies of informed consent will be available in the local language of the participants (French, Kiswahili or Chichewa) and where applicable, English. Data collectors will explain the informed consent form and respondents will be encouraged to ask for clarification or ask any questions. Respondents able to read and write will be required to sign the consent form themselves. In case of illiterate study participants, the data collectors will read the form carefully to them and they will countersign the form using their thumb impression. Where participants prefer to provide verbal consent in place of written consent, the consent will be audio-recorded. In this case, the participant or the researcher will read the paragraph

For peer review only

granting consent aloud while being audio-recorded. Interviews conducted under verbal consent can only proceed if there is at least an audio recording of the consent. The respondent can still decline audio recording for the full interview. Where the respondent does not give consent for audio recording, only handwritten notes will be taken during the interview.

Process of withdrawal from the study

The consent procedures make it clear to research participants that they have the right to withdraw from the study at any point, and to refuse to answer questions without any negative consequences. Possibilities for study withdrawal would include

- Where respondents have identified that they would be at risk from participating in the study
- Where a respondent self-reports that there has been a threat, verbal abuse or attempts to restrict their access to care or other services due to their participation in the data collection.
- When the research team identifies that there has been an incident, where recriminations have been levelled against respondents due to their participation in the study.
- Where a potential respondent declines providing any type of documented consent (written or verbal).

We received ethical approval from the local and national institutional review boards as follows: Karolinska Institutet, Sweden (Etikprövningsmyndigheten Dnr 2020–01587); Uganda National Council for Science and Technology (UNCST) (HS1324ES); Muhimbili University of Health And Allied Sciences (MUHAS) Research and Ethics Committee, Tanzania (MUHAS-REC-04-2020-118) and The Aga Khan University Ethical Review Committee, Tanzania (AKU/2019/044/fb); College of Medicine Research and Ethics Committee (COMREC), Malawi (COMREC P.04/20/3038); Comité National d'Ethique pour la Recherche en Santé, Cotonou, Bénin (83/MS/DC/SGM/CNERS/ST); The Institutional Review Board at the Institute of Tropical Medicine Antwerp and The Ethics Committee at the University Hospital Antwerp, Belgium, (ITG 1375/20. B3002020000116).

For peer review only

Dissemination

The results of this study will be published following the European Union's open-access policy. As mentioned above, we will use several avenues. First, we will organise workshops at hospital- district- and national level in the four study countries. Second, we will draft and submit scientific publications to open access journals and submit our work for presentation at conferences. Third, all publications and products will be published in a website (alert.ki.se).

For peer review only

Discussion

In implementation science, finding heterogeneity in results across different settings and types of organisations and by different actors is common. This variation may often be explained by the dynamic interplay between the intervention, its context, the implementation process and the agency of involved actors. When these interactions are not accounted for in an evaluation, the result is a 'black box' of knowledge that provides little or no explanatory insight on causal factors that explain how interventions lead to outcomes. In response to the evaluation approaches dominant in the 1970s and which focused almost exclusively on effectiveness, theory-driven inquiry methods were developed.[27] Theory-based evaluation[28], theories of change[29] and realist evaluation[20] all present methods to open the black box between intervention and outcome.

The 2015 guidance on the conduct of process evaluation developed by the UK Medical Research Council (MRC) calls for paying more attention to the intervention theory.[13] The updated guidance published in 2021 reinforced this and emphasised also the role of context.[30] This resonates with a similar shift in the field of implementation research, which is increasingly using conceptual models, frameworks and theories to design interventions but also to guide the design of evaluations, interpret findings and enhance translation of research into policy and practice.[31] Kislov *et al.* call for using mid-range theories of implementation, referring to Merton's definition of middle range theories as

theories that are situated between minor working hypotheses (nowadays often referred to as programme theories) and the all-inclusive unified theory (or grand theories).[32] Yet, the use of theory in process evaluations remains limited. In their recent systematic review of process evaluations embedded in trials, McIntyre and colleagues found that in about 66% of the reviewed papers, the authors stated to use theory. Yet only in 26% of the reviewed studies, theoretical propositions are actually applied, and in only 7% of the studies did the authors test theory.[33]

Previous studies have shown that realist principles can be adapted and applied to all phases of the PE cycle.[34] A realist process evaluation may offer two major advantages. First, it offers the possibility to systematically test the intervention theory by grounding it in the existing body of knowledge and testing it empirically, augmenting its explanatory power in the process.[35] Second, it allows to build up theories by accumulating empirical insights that push the programme theory to the level of a mid-range theory. Such mid-range theory has explanatory power extended to other settings, thereby offering the possibility to inform decisions on introducing or scaling up similar interventions.[18] Within the ALERT project, carrying out a realist process evaluation across eight hospitals in four countries will allow us not only to better understand the differential effects of the ALERT intervention in different hospitals in different countries, but also to contribute to the methodological developments within the field of process evaluations.

As with any study, there are some limitations to this study. Social desirability bias can occur when respondents answer to questions based on what they believe the interviewer wants to hear, or when specific responses are assumed to be more socially acceptable. Attempts to minimize this bias will be made by asking to view documentation where appropriate and triangulating data from multiple sources across work packages. The challenges of cross-cultural differences, power imbalances and western-dominated theories in realist research are additional recognised risks.[36] Approaches to mitigate this include the use of local researchers guided by a trained doctoral student with oversight

For peer review only

provided by a principal investigator who is well-versed in realist methodology; contextualisation and validation of data through participatory workshops and iterative meetings; and continuous reflection by evaluation team members on their positioning within the research study and potential sources of bias.

Contributors

IOA and BM designed the study, wrote the study protocol, designed the data collection guides and the analytical strategy, drafted the initial manuscript and drafted the final version. VCP, LB, CH and JPD contributed to the study design and the design of the data collection guides. IOA, BM, VCP, LB, CH, JPD, CH, CBM, SM, DM, GN and KSA contributed to the initial draft of the manuscript and to the final version.

Funding

This study is part of the ALERT project which is funded by the European Commission's Horizon 2020 (No 847824) under a call for Implementation research for maternal and child health. The funder had no role in the design of the study and collection, analysis, and interpretation of data and in writing the manuscript. The contents of this article are solely the responsibility of the authors and do not reflect the views of the European Union.

Competing interests

The authors declare that they have no competing interests.

Acknowledgments

We would like to acknowledge the support of the ALERT study team, which is a consortium of researchers and implementers of eight institutions across Europe, Benin, Malawi, Tanzania and Uganda. This group developed the ALERT project and is responsible for the implementation and the evaluation of the multifaceted intervention. The composition of the group is as follows.

For peer review only

The ALERT Study Team

Ahossi Angèle Florence Laure, Andrea B. Pembe, Ann-Beth Nygaard Moller, Antoinette Sognonvi, Armelle Vigan, Banougnin Bolade Hamed, Beatrice Mwilike, Kéfilath Bello, Bianca Kandeya, Christelle Boyi Metogni, Bruno Marchal, Claudia Hanson, Dickson Mkoka, Effie Chipeta, Elizabeth Ombeva Ayebare, Fadhlun M Alwy Al-Beity, Gertrude Namazzi, Gisele Houngbo, Gottfried Agballa, Hashim Hounkpatin, Helga Naburi, Helle Mölsted Alvesson, Hussein L. Kidanto, Jean-Paul Dossou, Joanne Welsh, Joseph Akuze, Josephine Babirye, Kristi Sidney Annerstedt, Lenka Benova, Lilian Mselle, Mechthild Gross, Muzdalifat Abeid, Nicola Orsini, Peter Waiswa, Philip Wanduru, Razak Mussa, Regine Unkels, Rian Snijders, Samuel Meja, Schadrac Agbla, Therese Delvaux, Tumbwene Mwansisya, Virginia Castellano Pleguezuelo, William Stones, Wim Van Damme, Yesaya Z. Nyirenda and Zamoyoni Julius.

Data and Safety Monitoring Board (DSMB)

Jennifer Hall, Erik Lampa, and Zahida Qureshi.

References

1. UN IGME. Levels & Trends in Child Mortality. Report 2020. New York: UN Inter-agency Group for Child Mortality Estimation (UN IGME), 2018.

2. GBD Child Mortality Collaborators. Global, regional, national, and selected subnational levels of stillbirths, neonatal, infant, and under-5 mortality, 1980-2015: a systematic analysis for the Global Burden of Disease Study 2015. *LANCET* 2015;388(10053):1725-74.

3. WHO. Trends in maternal mortality: 1990 to 2015 : estimates by WHO, UNICEF, UNFPA, World Bank Group and the United Nations Population Division. Geneva: World Health Organization, UNICEF, United Nations, Department of Economic and Social Affairs, Population Division, World Bank. , 2015.

4. WHO. WHO Recommendations Intrapartum care for a positive childbirth experience. Geneva: World Health Organization. , 2018.

5. Rowe AK, Rowe SY, Peters DH, et al. Effectiveness of strategies to improve health-care provider practices in low-income and middle-income countries: a systematic review. *Lancet Glob Health* 2018;6(11):e1163-e75. doi: 10.1016/S2214-109X(18)30398-X [published Online First: 2018/10/13]

6. Walker D, Otieno P, Butrick E, et al. Effect of a quality improvement package for intrapartum and immediate newborn care on fresh stillbirth and neonatal mortality among preterm and low-birthweight babies in Kenya and Uganda: a cluster-randomised facility-based trial. *Lancet Glob*

Health 2020;8(8):e1061-e70. doi: 10.1016/S2214-109X(20)30232-1 [published Online First: 2020/07/28]

7. Zamboni K, Singh S, Tyagi M, et al. Effect of collaborative quality improvement on stillbirths, neonatal mortality and newborn care practices in hospitals of Telangana and Andhra Pradesh, India: evidence from a quasi-experimental mixed-methods study. *Implement Sci* 2021;16(1):4. doi: 10.1186/s13012-020-01058-z [published Online First: 2021/01/09]

8. Zamboni K, Baker U, Tyagi M, et al. How and under what circumstances do quality improvement collaboratives lead to better outcomes? A systematic review. . *Implement Science* 2020;15(27)

9. Akuze J, Annerstedt KS, Benova L, et al. Action leveraging evidence to reduce perinatal mortality and morbidity (ALERT): study protocol for a stepped-wedge cluster-randomised trial in Benin, Malawi, Tanzania and Uganda. *BMC Health Serv Res* 2021;21(1):1324. doi: 10.1186/s12913-021-07155-z [published Online First: 20211211]

10. Punch K. Introduction to social research. London: SAGE Publications 2005.

11. Siddiqi K, Newell J, Robinson M. Getting evidence into practice: what works in developing countries. *International Journal for Quality in Health Care* 2005;17(5):447-54.

12. Grimshaw JM, Eccles MP, Lavis JN, et al. Knowledge translation of research findings. *Implement Sci* 2012;7:50. doi: 10.1186/1748-5908-7-50 [published Online First: 2012/06/02]

13. Moore G, Audray S, Barker M, et al. Process evaluation of complex interventions: Medical Research Council guidance. *BMJ* 2015;350(h1258)

14. Public Health England. Guidance. Process Evaluation London: Public Health England; 2018 [accessed 1 June 2021.

15. Marchal B, Kegels G, Van Belle S. Theory and realist methods. In: Emmel N, Greenhalgh J, Manzano A, et al., eds. Doing Realist Research SAGE London: Sage Publications 2018:79-90.

16. Westhorp G. Using complexity-consistent theory for evaluating complex systems. *Evaluation* 2012;18(4):405-20.

17. Marchal B, Van Belle S, De Brouwere V, et al. Complexity in health. Consequences for research and evaluation. FEMHealth Discussion Papers. Aberdeen: FEMHealth, 2014.

18. Pawson R. The science of evaluation: a realist manifesto. London: SAGE Publications 2013.

19. Wong G, Westhorp G, Manzano A, et al. RAMESES II reporting standards for realist evaluations. *BMC medicine* 2016;14(96)

20. Pawson R, Tilley N. Realistic Evaluation. London: Sage 1997.

21. Marchal B, Van Belle S, Van Olmen J, et al. Is realist evaluation keeping its promise? A literature review of methodological practice in health systems research. *Evaluation* 2012;18(2):192-212.

22. Kitson A, Harvey G, McCormack B. Enabling the implementation of evidence based practice: a conceptual framework. *Qual Health Care* 1998;7(3):149-58. doi: 10.1136/qshc.7.3.149 [published Online First: 1998/08/05]

23. Yin R. Case Study Research. Design and Methods. 5 ed. Los Angeles: SAGE 2014.

24. Mukumbang F, Van Belle S, Marchal B, et al. Unravelling how and why the Antiretroviral Adherence Club Intervention works (or not) in a public health facility: A realist explanatory theory-building case study. *Plos One* 2019;14(1):e0210565.

25. Spencer L, Ritchie J, O'Connor W. Carrying out Qualitative Analysis. ; 2003. p. In: Ritchie J, Lewis J, eds. Qualitative research practice: a guide for social science students and researchers. London: SAGE Publications 2003:219–62.

For peer review only

26. World Medical Association. WMA Declaration of Helsinki – Ethical principles for medical research involving human subjects. Available on: https://www.wma.net/policies-post/wma-declaration-of-helsinki-ethical-principles-for-medical-research-involving-human-subjects/. 2013

27. Rogers P. Theory-based evaluation: reflections ten years on. *New Directions for Evaluation* 2007;114(Summer):63-84.

28. Chen H, Rossi P. Evaluating with sense - The theory driven approach. *Evaluation Review* 1983;7:283-302.

29. Connell JP, Kubisch A, Schorr L, et al. New approaches to evaluating community initiatives. Concepts, methods, and contexts. Washington D.C.: The Aspen Institute 1995.

30. Skivington K, Matthews L, Simpson SA, et al. A new framework for developing and evaluating complex interventions: update of Medical Research Council guidance. *BMJ* 2021;374:n2061. doi: 10.1136/bmj.n2061 [published Online First: 20210930]

31. Kislov R, Pope C, Martin GP, et al. Harnessing the power of theorising in implementation science. *Implement Sci* 2019;14(1):103. doi: 10.1186/s13012-019-0957-4 [published Online First: 2019/12/12]

32. Merton RK. Social theory and social structure. New York: The Free Press 1968.

33. McIntyre S, Francis J, Gould N, et al. The use of theory in process evaluations conducted alongside randomized trials of implementation interventions: A systematic review. *Translational Behavioral Medicine* 2020;10:168-78.

34. Fletcher A, Jamal F, Moore G, et al. Realist complex intervention science: Applying realist principles across all phases of the Medical Research Council framework for developing and evaluating complex interventions. *Evaluation (Lond)* 2016;22(3):286-303. doi: 10.1177/1356389016652743 [published Online First: 2016/08/02]

35. Rycroft-Malone J, Seers K, Eldh AC, et al. A realist process evaluation within the Facilitating Implementation of Research Evidence (FIRE) cluster randomised controlled international trial: an exemplar. *Implementation Science* 2018;13(138)

36. Gilmore B. Realist evaluations in low- and middle-income countries: reflections and recommendations from the experiences of a foreign researcher. *BMJ Glob Health* 2019;4(5):e001638. doi: 10.1136/bmjgh-2019-001638 [published Online First: 2019/11/22]

FIGURE TITLES AND LEGENDS:

Figure 1. The ALERT intervention

Figure 2. Sequential integration of hospitals into the trial

Figure 3. The ALERT theory of change

For peer review only

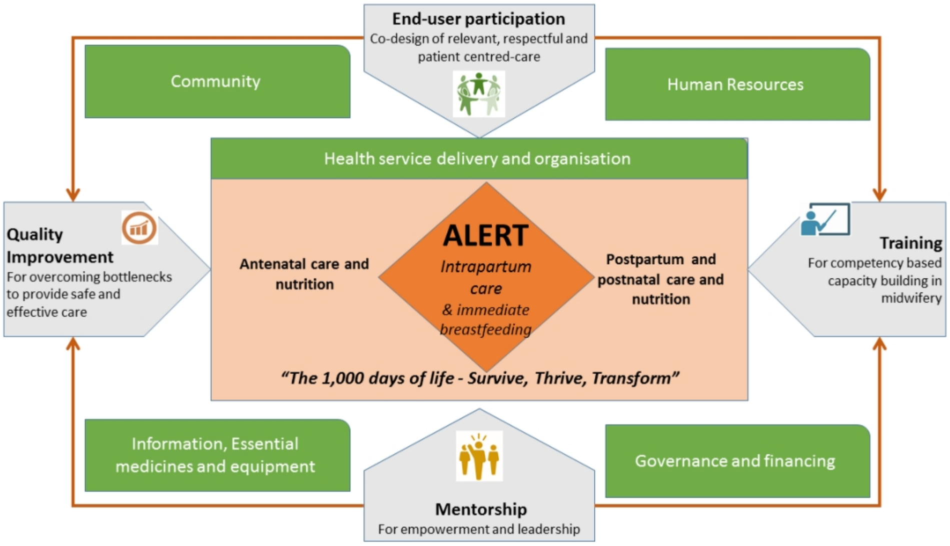

The ALERT intervention

338x190mm (96 x 96 DPI)

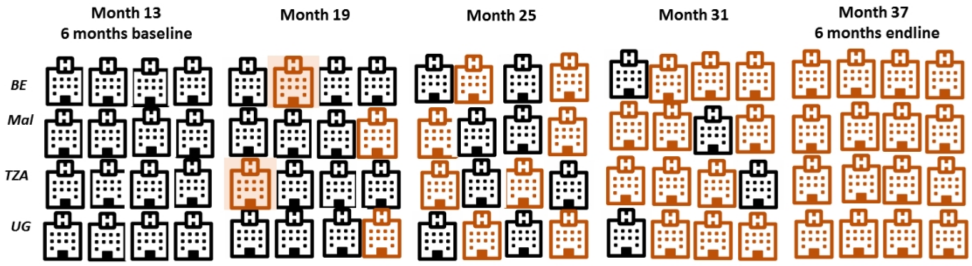

Sequential integration of hospitals into the trial

409x190mm (96 x 96 DPI)

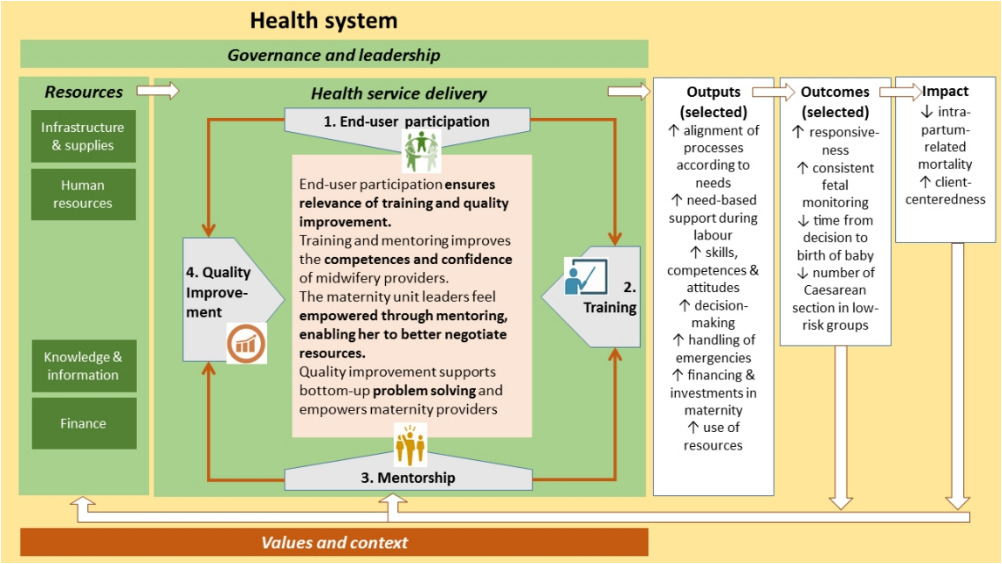

338x190mm (96 x 96 DPI)

Additional File 1

Characteristics of the study countries

	Benin	Malawi	Tanzania	Uganda
Country-level indicators				
Estimated population (in 2020, million)	12.1	20.3	62.8	47.2
Maternal mortality ratio per 100,000 live births (2015) [3]	405	634	398	343
Neonatal mortality rate per 1,000 live births (2018) [1]	32.7	22.7	21.1	20.2
Stillbirth rate per 1,000 total births (2015) [102]	30.3	21.8	22.4	21.0
% of births in health facilities (DHS Statcompiler) #	86.9%	91.4%	62.6%	73.4%
Annual growth rate in % of births in health facilities #	1.3%	3.2%	2.6%	4.4%
% of facility births in hospitals #	55.1%	42.2%	47.8%	47.8%
% of all live births by caesarean section # / Poorest v Richest wealth quintile	5.4%<br>2.7% - 11.5%	6.1%<br>3.0% - 9.1%	5.9%<br>2.4% - 15.8%	6.2%<br>2.7% - 14.2%
Among live births in health facilities				
% of women checked by a health professional before discharge from facility following childbirth #	68%	57%	51%	47%
% of all live births by caesarean section #	6.3%	6.3%	9.5%	8.3%
Among live births in hospitals				
Neonatal mortality per 1,000 live births #	20.6	35.8	31.8	27.1
% of newborns breastfed within 1 hour of birth #	67.8%	73.1%	54.9%	65.3%
Health system indicators				
Doctors /10,000 people population ^	1.6 (2016)	1.2 (2016)	0.4 (2014)	0.9 (2015)
Nursing cadres /10,000 people population ^	6.2 (2016)	2.5 (2016)	4.1 (2014)	6.3 (2015)
Predominant midwifery provider [103]	Midwife	Nurse-midwife	Nurse-midwife	Midwife
Hospital beds / 10,000 people population ^	5 (2010)	13 (2011)	7 (2010)	5 (2010)
Current health expenditure per capita (USD PPP, 2015) ^	85	108	97	139
Out-of-pocket expenditure as % of current health expenditure (2015?)^	41	11	26	41
User fees for childbirth (vaginal/caesarean) ^	Official fees	No official	No official	No official

additional analysis of Demographic and Health Survey data, Benin; 2011-12; Malawi: 2015-16; Tanzania: 2015-16; Uganda: 2016
^WHO observer http://apps.who.int/gho/data/node.main.HWFGRP_0020?lang=en

For peer review only

For peer review only

Additional File 2- Rameses checklist applied to our protocol

The checklist presented below is drawn from Wong G, Westhorp G, Manzano A, Greenhalgh J, Jagosh J, Greenhalgh T: RAMESES II reporting standards for realist evaluations. BMC Medicine 2016, 14(96). Please note that this guidance was developed for peer reviewers of realist evaluations and not for relaits evaluation protocols.

			**Reported in document Y/N/Unclear**	**Page(s) in document**
TITLE				
1		In the title, identify the document as a realist evaluation	Yes	*Title*
SUMMARY OR ABSTRACT				
2		Journal articles will usually require an abstract, while reports and other forms of publication will usually benefit from a short summary. The abstract or summary should include brief details on: the policy, programme or initiative under evaluation; programme setting; purpose of the evaluation; evaluation question(s) and/or objective(s); evaluation strategy; data collection, documentation and analysis methods; key findings and conclusions<br>Where journals require it and the nature of the study is appropriate, brief details of respondents to the evaluation and recruitment and sampling processes may also be included<br>Sufficient detail should be provided to identify that a realist approach was used and that realist programme theory was developed and/or refined	Yes	*Abstract*
INTRODUCTION				

For peer review only - http://bmjopen.bmj.com/site/about/guidelines.xhtml

3	Rationale for evaluation	Explain the purpose of the evaluation and the implications for its focus and design	Yes	*Introduction*, paragraph 5 and 6

			**Reported in document Y/N/Unclear**	**Page(s) in document**
4	Programme theory	Describe the initial programme theory (or theories) that underpin the programme, policy or initiative	Yes	We present how the initial programme theory will be developed in Step 1 of the study (in section *Methods and analysis – Objective 1: Defining the Initial Programme Theory*).
5	Evaluation questions, objectives and focus	State the evaluation question(s) and specify the objectives for the evaluation. Describe whether and how the programme theory was used to define the scope and focus of the evaluation	Yes	In the section *Methods and analysis – Objectives,* we present the study objectives, which are structured along the RE rationale.
6	Ethical approval	State whether the realist evaluation required and has gained ethical approval from the relevant authorities, providing details as appropriate. If ethical approval was deemed unnecessary, explain why	Yes	At the end of the section *Ethics and dissemination*, we present the approvals of the local and national institutional review boards

For peer review only

METHODS				
7	Rationale for using realist evaluation	Explain why a realist evaluation approach was chosen and (if relevant) adapted	Yes	*Introduction,* paragraph 7 and

			**Reported in document Y/N/Unclear**	**Page(s) in document**
				Methods and analysis section, paragraph 1.
8	Environment surrounding the evaluation	Describe the environment in which the evaluation took place	Yes	*Introduction*, paragraph 1-4
9	Describe the programme policy, initiative or product evaluated	Provide relevant details on the programme, policy or initiative evaluated	Yes	*Introduction*, paragraph 3 and 4
10	Describe and justify the evaluation design	A description and justification of the evaluation design (i.e. the account of what was planned, done and why) should be included, at least in summary form or as an appendix, in the document which presents the main findings. If this is not done, the omission should be justified and a reference or link to the evaluation design given. It may also be useful to publish or make freely available (e.g. online on a website) any original evaluation design document or protocol, where they exist	Yes	*Introduction*, paragraph 5 and 6

For peer review only

11	Data collection methods	Describe and justify the data collection methods – which ones were used, why and how they fed into developing, supporting, refuting or refining programme theory<br>Provide details of the steps taken to enhance the trustworthiness of data collection and documentation	Yes	Section *Methods and analysis - Data collection*, . Table 1 shows the relation between study objectives, methods and product/finality.<br>See also Section *Data collection* tools and *Data management*

			Reported in document Y/N/Unclear	Page(s) in document
12	Recruitment process and sampling strategy	Describe how respondents to the evaluation were recruited or engaged and how the sample contributed to the development, support, refutation or refinement of programme theory	Yes	Section *Methods and analysis - Data collection*.
13	Data analysis	Describe in detail how data were analysed. This section should include information on the constructs that were identified, the process of analysis, how the programme theory was further developed, supported, refuted and refined, and (where relevant) how analysis changed as the evaluation unfolded	Yes	Section *Methods and analysis - Data analysis*
RESULTS				

For peer review only - http://bmjopen.bmj.com/site/about/guidelines.xhtml

14	Details of participants	Report (if applicable) who took part in the evaluation, the details of the data they provided and how the data was used to develop, support, refute or refine programme theory	No	Not applicable
15	Main findings	Present the key findings, linking them to contexts, mechanisms and outcome configurations. Show how they were used to further develop, test or refine the programme theory	No	Not applicable
DISCUSSION				
16	Summary of findings	Summarise the main findings with attention to the evaluation questions, purpose of the evaluation, programme theory and intended audience	No	Not applicable
17	Strengths, limitations and future directions	Discuss both the strengths of the evaluation and its limitations. These should include (but need not be limited to): (1) consideration of all the steps in the evaluation processes; and (2) comment on the adequacy, trustworthiness and value of the explanatory insights which emerged	Yes	Section Discussion, paragraph 4
			**Reported in document Y/N/Unclear**	**Page(s) in document**
		In many evaluations, there will be an expectation to provide guidance on future directions for the programme, policy or initiative, its implementation and/or design. The particular implications arising from the realist nature of the findings should be reflected in these discussions		
18	Comparison with existing literature	Where appropriate, compare and contrast the evaluation's findings with the existing literature on similar programmes, policies or initiatives	No	Not applicable

For peer review only - http://bmjopen.bmj.com/site/about/guidelines.xhtml

19	Conclusion and recommendations	List the main conclusions that are justified by the analyses of the data. If appropriate, offer recommendations consistent with a realist approach	No	Not applicable
20	Funding and conflict of interest	State the funding source (if any) for the evaluation, the role played by the funder (if any) and any conflicts of interests of the evaluators	Yes	See *Funding statement*

For peer review only

For peer review only - http://bmjopen.bmj.com/site/about/guidelines.xhtml
